# Decreased GLUT2 and glucose uptake contribute to insulin secretion defects in MODY3/HNF1A hiPSC-derived mutant β cells

**DOI:** 10.1038/s41467-021-22843-4

**Published:** 2021-05-25

**Authors:** Blaise Su Jun Low, Chang Siang Lim, Shirley Suet Lee Ding, Yaw Sing Tan, Natasha Hui Jin Ng, Vidhya Gomathi Krishnan, Su Fen Ang, Claire Wen Ying Neo, Chandra S. Verma, Shawn Hoon, Su Chi Lim, E. Shyong Tai, Adrian Kee Keong Teo

**Affiliations:** 1 Stem Cells and Diabetes Laboratory, Institute of Molecular and Cell Biology (IMCB), Agency for Science, Technology and Research (A*STAR), Singapore, Singapore; 2 Yong Loo Lin School of Medicine, National University of Singapore, Singapore, Singapore; 3 Saw Swee Hock School of Public Health, National University of Singapore, Singapore, Singapore; 4 Bioinformatics Institute, Agency for Science, Technology and Research (A*STAR), Singapore, Singapore; 5 Molecular Engineering Lab (MEL), IMCB, A*STAR, Singapore, Singapore; 6 Khoo Teck Puat Hospital, Singapore, Singapore; 7 Department of Biological Sciences, National University of Singapore, Singapore, Singapore; 8 School of Biological Sciences, Nanyang Technological University, Singapore, Singapore

**Keywords:** Induced pluripotent stem cells, Stem-cell differentiation, Diabetes, Endocrine system and metabolic diseases

## Abstract

Heterozygous *HNF1A* gene mutations can cause maturity onset diabetes of the young 3 (MODY3), characterized by insulin secretion defects. However, specific mechanisms of MODY3 in humans remain unclear due to lack of access to diseased human pancreatic cells. Here, we utilize MODY3 patient-derived human induced pluripotent stem cells (hiPSCs) to study the effect(s) of a causal *HNF1A*^*+/H126D*^ mutation on pancreatic function. Molecular dynamics simulations predict that the H126D mutation could compromise DNA binding and gene target transcription. Genome-wide RNA-Seq and ChIP-Seq analyses on MODY3 hiPSC-derived endocrine progenitors reveal numerous HNF1A gene targets affected by the mutation. We find decreased glucose transporter GLUT2 expression, which is associated with reduced glucose uptake and ATP production in the MODY3 hiPSC-derived β-like cells. Overall, our findings reveal the importance of HNF1A in regulating *GLUT2* and several genes involved in insulin secretion that can account for the insulin secretory defect clinically observed in MODY3 patients.

## Abstract (web summary)

Heterozygous HNF1A mutations can give rise to maturity onset diabetes of the young 3 (MODY3), characterized by insulin secretion defects. Here the authors show that MODY3-related HNF1A mutation in patient hiPSCderived pancreatic cells decreases glucose transporter GLUT2 expression due to compromised DNA binding.

## Introduction

Maturity onset diabetes of the young 3 (MODY3) is the most common type of monogenic diabetes and is typically characterized by an early onset before 25 years of age. MODY3 patients are known to suffer from progressive pancreatic β cell failure that culminates in a loss of insulin secretory function^1^. MODY3 is caused by mutations in the *HNF1A* gene^2,3^. The human *HNF1A* gene codes for a transcription factor that consists of an N-terminal dimerization domain, a homeobox DNA-binding domain and a C-terminal transactivation domain^3,4^. HNF1A is expressed in several organs including the liver, kidney, pancreas and intestine^5,6^.

Due to the lack of access to human pancreatic cells that are undergoing development and maturation into β cells, early studies of MODY3 were conducted using rodent models. In rodent models, insulin secretory defects and higher blood glucose concentrations are found to occur only in *Hnf1α*^*−/−*^ homozygous knockout mice but not in *Hnf1α*^*+/−*^ heterozygous mice^7^. This is in contrast to the MODY3 pathology in humans whereby all known cases are caused by autosomal dominant *HNF1A*^*+/−*^ heterozygous mutations^8^. This highlights the importance of studying the effects of heterozygous HNF1A mutations in human models.

Recently, Haliyur et al. (2018) reported a *HNF1A*^*+/T260M*^ variant found in diabetic human islets that affected the transcriptional regulatory networks required for β cell function^9^. The *HNF1A*^*+/T260M*^ β cells exhibited a decreased expression of genes involved in glucose metabolism, ATP production, gene transcription, intracellular protein transport, cell stress response and cell signaling. However, human islets harboring *HNF1A* mutations are scarce. To further explore the roles of *HNF1A*, Cardenas-Diaz et al. (2019) reported the use of CRISPR/Cas9-mediated *HNF1A*^*+/−*^ and *HNF1A*^*−/−*^ allelic series in a human embryonic stem-cell (hESC) differentiation model^10^. The study revealed that *HNF1A* deletion resulted in pancreatic developmental and metabolic defects, as well as dysregulation of glycolysis and mitochondrial respiration. While these studies have implicated *HNF1A* mutations in reduced ATP production and defective mitochondrial respiration, the effects of *HNF1A* mutations on the rest of the stimulus-secretion coupling pathway—glucose uptake, K_ATP_ channel and voltage-gated Ca^2+^ signaling—in human β cells remain largely unexplored. Furthermore, the effects of a patient-specific *HNF1A* heterozygous MODY3 mutation on HNF1A transcriptional targets and insulin secretion still remain largely unclear.

In this work, we leverage on MODY3-hiPSC disease modeling by generating patient-specific induced pluripotent stem-cell lines (hiPSCs) from two Singaporean MODY3 patients harboring a unique *HNF1A*^*+/H126D*^ mutation^11^, and differentiate them towards the pancreatic lineage in order to study its effect on insulin secretion in human pancreatic β cells. We find that the *HNF1A* mutation results in a loss of binding and decreased expression of genes involved in pancreas development, β cell survival, insulin secretion, insulin resistance and type 2 diabetes (T2D). In addition, we comprehensively demonstrate that the *HNF1A* mutation causes GLUT2 deficiency that is associated with reduced glucose uptake and ATP production. This may partly account for the lack of insulin secretion in MODY3 patients. We propose that a modulation of glucose uptake or ADP to ATP conversion may be a viable means to reinstate the insulin secretory capacity of these dysfunctional β cells.

## Results

### HNF1A H126D mutation altered amino acid interactions

**Fig. 1:**
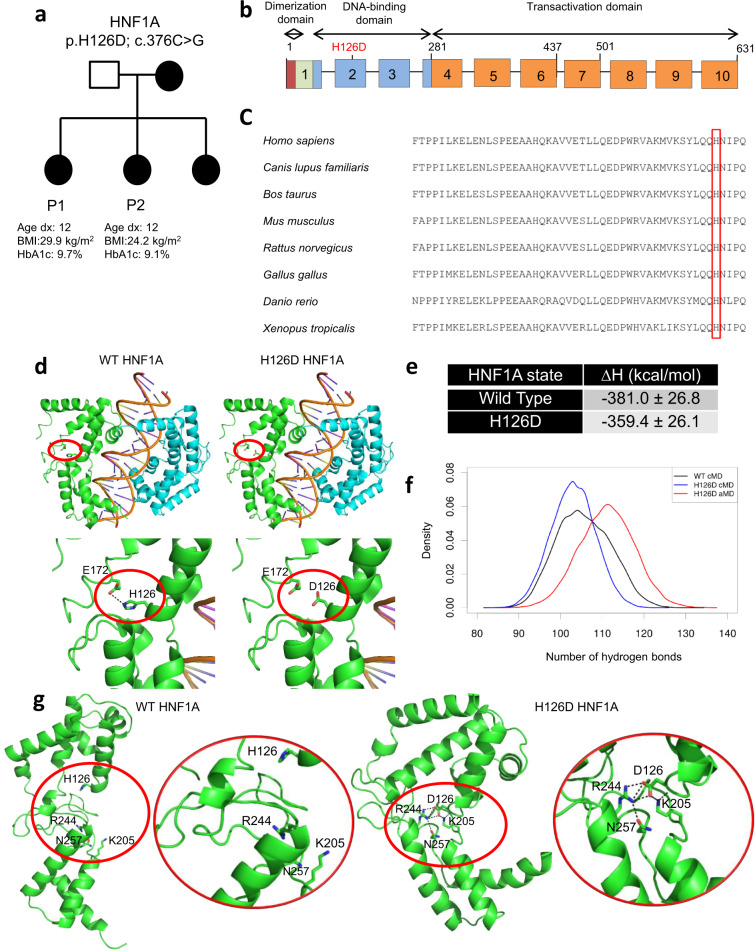
HNF1A H126D mutation alters amino acid interactions and HNF1A protein conformation. **a** MODY3 family pedigree. Square denotes male, circles denote females. Solid circles denote diabetic individuals. BMI body mass index, HbA1c haemoglobin A1c. **b** *HNF1A* gene structure consists of three functional domains: dimerization domain (red), DNA-binding domain (blue) and transactivation domain (orange). H126D mutation occurs in the DNA-binding domain. **c** Multiple sequence alignment of HNF1A protein sequences amongst various species. H126 is highlighted in red box. **d** Structures of WT HNF1A (PDB 1IC8) and H126D mutant protein (green and cyan) bound to a gene promoter. Interactions between H126/D126 and E172 are highlighted in red circles. **e** Computed binding free energies (∆H) for formation of protein–DNA complexes for WT HNF1A and H126D mutant (entropy change was assumed to be the same for all complexes and therefore ignored). **f** Histogram of intramolecular hydrogen bond counts obtained from conventional molecular dynamics (cMD) simulations of WT (black) and mutant HNF1A H126D (blue) complexed with DNA, and accelerated molecular dynamics (aMD) simulations of H126D mutant HNF1A complexed with DNA (unweighted, red). A hydrogen bond is counted when the angle between the donor and acceptor heavy atoms is greater than or equal to 135° and their distance is less than or equal to 3.5 Å. **g** Representative trajectory structures obtained from the cMD simulations of WT and mutant H126D HNF1A. The insets (highlighted in red circles) show the interactions of H126 and D126 with neighboring residues. WT wild-type, P1 patient 1, P2 patient 2. “See also Supplementary Movies 1 and 2, and Fig. S1.” Source data are provided as a Source data file.

We recruited a pair of Singaporean sisters (labelled P1 and P2, respectively) diagnosed with MODY3 at the age of 12 (Fig. 1a), caused by a unique H126D (c.376 C > G) mutation that occurs in the DNA-binding domain of HNF1A^11^ (Fig. 1b). Multiple sequence alignment analysis of the HNF1A protein sequence revealed that H126 is a highly conserved amino acid across various species (Fig. 1c), suggesting that this amino acid may be critical for HNF1A protein function.

We first performed computational modeling studies to understand the impact of H126D mutation on the structure of unbound HNF1A and the interactions of HNF1A with DNA. The crystal structure of wild-type (WT) HNF1A bound to DNA^12^ shows that H126 forms a hydrogen bond with E172 at the interdomain region (Fig. 1d). Mutation of H126 to D126 is likely to result in electrostatic repulsion between the negatively charged residues D126 and E172, which could perturb the interdomain interactions and structure of HNF1A, thus affecting its ability to bind to DNA (Fig. 1d).

We performed molecular dynamics (MD) simulations of the complexes of WT and mutant HNF1A H126D with DNA and computed binding free energies for their formation based on their trajectory structures (Fig. 1e). There was no significant difference in the binding free energies for the WT and mutant proteins (Fig. 1e). We further performed accelerated MD (aMD) simulations to enhance conformational sampling of the mutant HNF1A–DNA complex. aMD is an enhanced sampling technique that applies a boost potential to lower the energy barriers of the system and accelerate conformational transitions. The final mutant HNF1A H126D–DNA complex structures from the conventional MD (cMD) simulations were used to initiate the aMD simulations, which were then run for an additional 200 ns. The mutant HNF1A remained bound to DNA in these extended simulations with no major secondary structure changes. There was an apparent stabilization of the mutant complex observed towards the end of the aMD simulations, as evidenced by the higher number of HNF1A–DNA hydrogen bond counts compared to both WT and mutant complexes in the cMD simulations (Fig. 1f). This is somewhat borne out by the formation of new salt bridges by D126 with K169 and/or R244 in all the mutant complex simulations (Fig. S1). Based on these initial results, it would seem that the H126D mutation has negligible effect on the structure of the HNF1A–DNA complex. However, as the mutant complex simulations were initiated from the bound state, they were unable to account for structural effects of the mutation on HNF1A prior to DNA binding. The structure of unbound HNF1A could be very different from its DNA-bound state.

To fully investigate the effect of the mutation on HNF1A, MD simulations of unbound WT and H126D mutant HNF1A were performed. We observed a drastic change in the conformation of WT HNF1A in the majority (4 out of 5) of the simulation runs (Fig. 1g and Supplementary Movie 1). WT HNF1A is highly flexible, with the homeodomain (POU_H_) and POU_s_ domain pulling away from each other, which causes the protein to adopt an open conformation. This allows K205, which is known to interact directly with DNA^12^, to be exposed and available for binding to DNA. In contrast, mutant HNF1A H126D is highly rigid and adopts a closed conformation in all simulation runs, as the POU_H_ and POU_S_ domains are held together by salt bridges and hydrogen bonds formed within a polar tetrad core of interdomain residues, namely D126, K205, R244 and N257 (Fig. 1g and Supplementary Movie 2). This increased rigidity and inability to adopt an open conformation could hamper induced-fit binding of mutant HNF1A H126D to DNA. D126 is also observed to form a salt bridge with K205 in the simulations, thus sequestering the latter within the interdomain region and rendering it inaccessible for interacting with DNA (Fig. 1g). Together, these effects are expected to reduce the binding affinity of mutant HNF1A H126D for DNA, which could interfere with the ability of the mutant protein to regulate gene expression.

### MODY3-hiPSCs as a human model to study pancreatic β cells

To investigate the effect of the HNF1A H126D mutation on the development and function of pancreatic β cells, we proceeded to establish a human patient-relevant model for MODY3 disease modeling. We obtained skin fibroblasts from the two MODY3 patients (P1 and P2) and generated three independent hiPSC lines from each of them (iP1a, iP1b, iP1c, iP2a, iP2b and iP2c) (Fig. S2) using episomal reprogramming^13^. These hiPSCs stained positively for pluripotency markers OCT4, SOX2, NANOG, SSEA4 and TRA-1–60 (Fig. S2a), formed the three germ layers in a teratoma assay (Fig. S2b), were karyotypically normal (Fig. S2c), and were sequenced to confirm their *HNF1A* c.376 C > G (H126D) genotype (Fig. S2d). Subsequently, we used H9 hESCs and iAGb (hiPSCs reprogrammed from a healthy donor)^14^ as two independent WT hPSC controls in this study.

### Downregulation of genes in β cell function

To model and study MODY3 in human pancreatic β cells, we then differentiated H9, iAGb and the patient-specific hiPSCs into pancreatic β-like cells using a published protocol^15^ (Figs. S3a and S3b). We verified the presence of C-peptide^+^ β-like cells (Fig. S3c) at the end of the differentiation and observed increased *INS* transcript expression throughout the course of differentiation (Fig. S3d). There were no distinct morphological differences between the two independent WT and six independent mutant β-like cells (Fig. S3e).

**Fig. 2:**
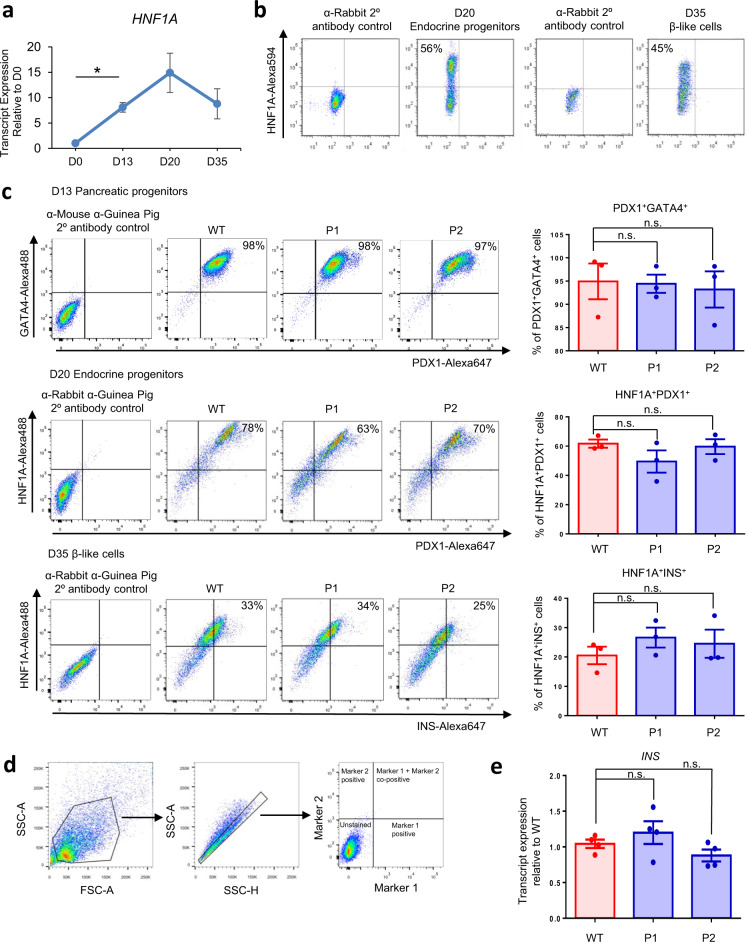
No differences in pancreatic differentiation efficiencies between WT and mutant cell lines. **a** RT-qPCR analysis of *HNF1A* transcripts during various stages of pancreatic differentiation. *n* = 3 independent experiments; *p* = 0.0173. **b** Flow cytometry analysis of HNF1A protein expression in differentiated endocrine progenitors and β-like cells. **c** Flow cytometry analysis of GATA4 and PDX1 protein expression in D13 pancreatic progenitors, HNF1A and PDX1 in D20 endocrine progenitors and HNF1A and INS in D35 β-like cells in WT (red) and mutant (blue) cell lines. *n* = 3 independent experiments. **d** Gating strategy used for flow cytometry for gating hPSC-derived pancreatic cells across various linages. The same gating strategy was used for all flow cytometry experiments presented in this paper (Figs. 2b, 2c and S2f). **e** RT-qPCR analysis of *INS* transcripts in D35 β-like cells. *n* = 4 independent experiments. D day of differentiation. WT wild-type, P1 patient 1, P2 patient 2. RT-qPCR quantitative reverse transcription polymerase chain reaction. For all statistical analysis: Error bars represent standard error of mean (SEM). Unpaired one-tailed Student’s *t*-test was performed. Asterisk indicates *P*-value < 0.05. n.s. non-significant. “See also Fig. S3.” Source data are provided as a Source data file.

We first evaluated the expression profile of *HNF1A* transcripts (Fig. 2a) and protein (Fig. 2b) during pancreatic β cell differentiation, and observed that HNF1A transcript and protein expression peaks on day 20 of the differentiation—the endocrine progenitor stage (Figs. 2a and 2b). To evaluate the pancreatic differentiation efficiencies of the WT and MODY3 hPSCs, we performed flow cytometry and immunostaining at various stages of the pancreatic differentiation on day 13 (pancreatic progenitors), day 20 (endocrine progenitors) and day 35 (β-like cells) (Figs. 2c, 2d, S3f, S4a and S4b). The flow cytometry analyses revealed that the WT, P1 and P2 lines generated >90% GATA4^+^PDX1^+^ pancreatic progenitors, >60% HNF1A^+^PDX1^+^ endocrine progenitors and >20% HNF1A^+^INS^+^ and HNF1A^+^NKX6.1^+^ β-like cells on day 13, day 20 and day 35 of the differentiation process respectively (Figs. 2c, 2d and S3f). There was no significant difference in the percentage of GATA4^+^PDX1^+^ pancreatic progenitors, HNF1A^+^PDX1^+^ endocrine progenitors and HNF1A^+^INS^+^ β-like cells generated from the MODY3-hiPSCs compared to the WT hiPSCs. Similarly, there was no significant difference in the *INS* transcript expression between the WT and mutant β-like cells (Fig. 2e). This suggests that the H126D mutation neither affects the formation of human β cells nor alters insulin production.

**Fig. 3:**
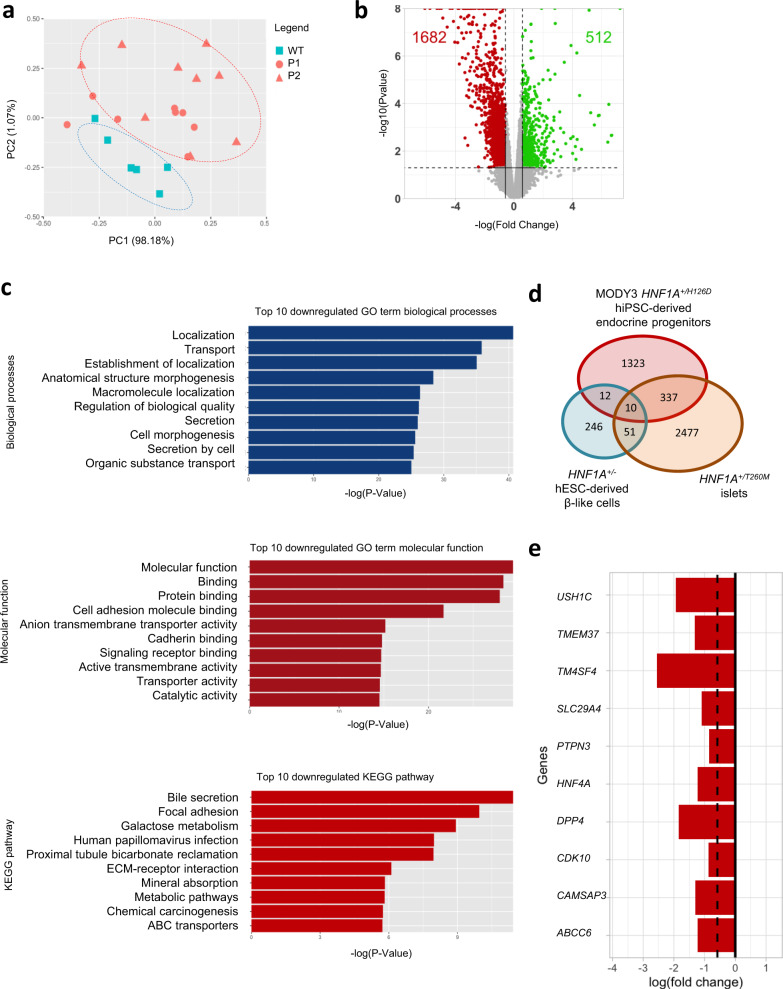
RNA-Seq analyses reveal a downregulation of genes involved in pancreatic endocrine and β cell function in MODY3 endocrine progenitors. **a** Principal component analysis (PCA) plot of RNA-Seq data for WT and MODY3-hPSC-derived day 20 endocrine progenitors. Each dot in the PCA plot represents an independently sampled replicate of each cell line. All the dots represent: WT (blue square) = H9 (3 replicates), iAGb (3 replicates), P1 (red circle) = P1a (2 replicates), P1b (3 replicates), P1c (3 replicates), P2 (red triangle) = P2a (3 replicates), P2b (3 replicates), P2c (3 replicates). PC principal component. **b** Volcano plot depicting differential gene expression (False discovery rate < 0.05 and Fold change > ±1.5 fold) in WT and mutant endocrine progenitors. Red dots represent downregulated genes and green dots indicate upregulated genes in mutant endocrine progenitors. **c** Gene Ontology (GO) and Kyoto Encyclopaedia of Genes and Genome (KEGG) analyses of top ten downregulated biological processes, molecular functions and KEGG pathways in mutant endocrine progenitors. Unadjusted *P*-values were calculated using two-sided Mann-Whitney U test according to the GOSeq protocol^97^. **d** Venn diagram depicting the number of genes that are downregulated in *HNF1A*^*+/H126D*^ endocrine progenitors, *HNF1A*^*+/T260M*^ islets and *HNF1A*^*+/−*^ hESC-derived β-like cells. Overlaps show the number of common downregulated genes. **e** Common genes downregulated in *HNF1A*^*+/H126D*^ endocrine progenitors, *HNF1A*^*+/T260M*^ islets, and *HNF1A*^*+/−*^ hESC-derived β-like cells. WT wild-type, P1 patient 1, P2 patient 2. hPSC human pluripotent stem cells. hESC human embryonic stem cells. “See also Fig. S5.”.

To evaluate the impact of *HNF1A*^*+/H126D*^ mutation on pancreatic β cell development, we performed RNA-Seq analyses on the endocrine progenitors, which maximally expressed HNF1A protein. We observed a distinct difference in the global transcriptomic expression profile between WT and *HNF1A*^*+/H126D*^ mutant cells as demonstrated by the separate clustering of WT cells from the mutant cells in the principal component analysis (PCA) plot (Fig. 3a). A total of 2194 genes were differentially expressed in the mutant cells, of which 1682 genes were downregulated and 512 genes were upregulated in mutants (Fig. 3b, Supplementary Data 1 and 2). HNF1A is known to function as a transcription activator that binds and promotes the expression of its target genes^16,17^. HNF1A has not been reported to function as a transcription repressor that exerts direct repressor effects on its targets, and genes that are repressed by HNF1A are typically indirect targets^18,19^. Hence, the upregulation of genes in the MODY3 mutants are likely indirect effects of the HNF1A H126D mutation, while genes that are downregulated in the mutants are potential direct targets of HNF1A perturbed by the HNF1A H126D mutation. Gene ontology (GO) analyses revealed that the downregulated genes were largely involved in biological processes such as localization, transport and secretion and molecular functions, such as protein binding and transporter activity (Fig. 3c).

Furthermore, we compared the genes downregulated in our *HNF1A*^*+/H126D*^ endocrine progenitors to genes that were recently found to be downregulated in *HNF1A*^*+/T260M*^ islets^9^ and in *HNF1A*^*+/−*^ hESC-derived β-like cells^10^ (Fig. 3d). We found 347 genes that were commonly downregulated in both *HNF1A*^*+/H126D*^ endocrine progenitors and *HNF1A*^*+/T260M*^ islets (Supplementary Data 3), while 22 genes were commonly downregulated in both *HNF1A*^*+/H126D*^ endocrine progenitors and *HNF1A*^*+/−*^ hESC-derived β cells (Supplementary Data 4). Ten genes were found to be downregulated in all *HNF1A*^*+/H126D*^ endocrine progenitors, *HNF1A*^*+/T260M*^ islets and *HNF1A*^*+/−*^ hESC-derived β cells (Fig. 3e). Several of the commonly downregulated genes in the *HNF1A*^*+/H126D*^ endocrine progenitors and *HNF1A*^*+/T260M*^ islets are involved in insulin secretion, glucose metabolism and MODY (Figs. S5a–d), suggesting that the downregulation of genes may be involved in the pathogenesis of MODY3 caused by HNF1A mutations located in the DNA-binding domain. Together, these analyses have identified a set of genes that are altered in human MODY3 pancreatic cells that could play important roles in mediating insulin secretion by the β cells.

### Putative HNF1A targets affected by *HNF1A*^*+/H126D*^ mutation

**Fig. 4:**
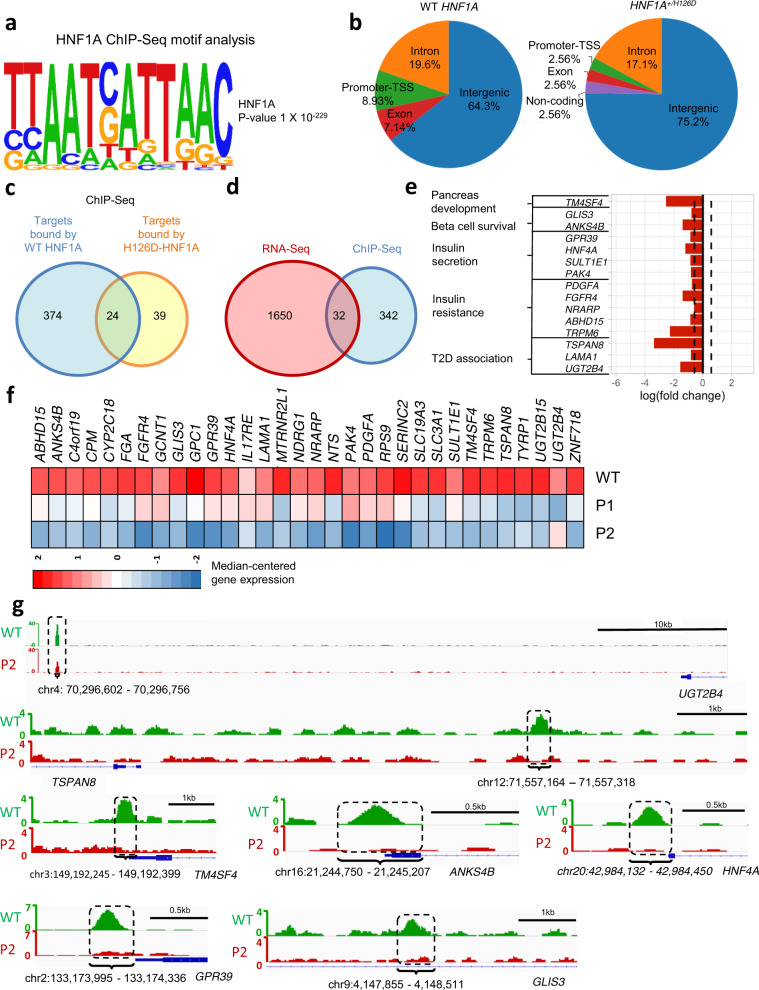
RNA-Seq and ChIP-Seq analyses reveal putative HNF1A targets affected by *HNF1A*^*+/H126D*^ mutation. **a** HNF1A consensus motif identified in our ChIP-Seq dataset. **b** Pie chart of global enrichment profile in ChIP-Seq analysis of WT and mutant endocrine progenitors. Venn diagram depicting **c** the number of targets bound (*P*-value < 1e-04 and >4 fold enrichment over input) by WT and mutant HNF1A in endocrine progenitors via ChIP-Seq analysis and, **d** the overlap of downregulated differentially expressed genes in mutant endocrine progenitors identified by RNA-Seq and ChIP-Seq targets enriched (*P*-value < 1e-04 and >4 fold enrichment over input) in WT HNF1A but not H126D. **e** Candidate HNF1A-bound genes that are downregulated in mutant endocrine progenitors. **f** Heatmap of genes that are both downregulated in RNA-Seq and ChIP-Seq analyses in mutant endocrine progenitors. The colour spectrum indicates relative expression levels within the samples (WT, P1 and P2), where red indicates relatively high expression, blue indicates relatively low expression. **g** Histogram showing ChIP-Seq enrichment regions. Regions where there is a difference in binding between WT (green) and P2 (*HNF1A*^*+/H126D*^) (red) are highlighted in dotted-line box with the nearest coding gene shown. ChIP-Seq Chromatin immunoprecipitation sequencing. WT wild-type. P1 patient 1, P2 patient 2.

To then determine the direct targets of HNF1A that could be responsible for β cell dysfunction in our MODY3 patients with *HNF1A*^*+/H126D*^ mutation, we performed ChIP-Seq analyses to compare targets bound by WT HNF1A versus H126D mutant protein in the differentiated endocrine progenitors. First, we were able to identify the HNF1A-binding motif (TTAATC/GATTAAC) in the HNF1A-bound regions (Fig. 4a). The HNF1A-binding motif was the top-enriched motif in the WT endocrine progenitors, and was not detected in the *HNF1A*^*+/H126D*^ endocrine progenitors (Figs. S6a and S6b). Our analyses revealed that there was a reduction in the promoter-binding activity by heterozygous HNF1A H126D mutant as compared to WT HNF1A protein (Fig. 4b). Overall, we found 374 targets to be bound by WT HNF1A but not by HNF1A H126D mutant protein (Fig. 4c and Supplementary Data 5).

Next, we overlapped the unbound targets identified in our HNF1A H126D ChIP-Seq with our RNA-Seq data that are both relevant for our MODY3 patients. We identified a total of 32 downregulated genes that are direct targets of HNF1A (Figs. 4d–f), of which 15 are known to be important for pancreas development and function (Fig. 4e). These included genes involved in pancreas development (*TM4SF4*)^20^, β cell survival (*GLIS3* and *ANKS4B*)^21–23^, insulin secretion (*GPR39, HNF4A, SULT1E1, PAK4*)^24–29^, insulin resistance (*PDGFA, FGFR4, NRARP, ABHD15* and *TRPM6*)^30–34^ and T2D (*TSPAN8, LAMA1* and *UGT2B4*)^35–38^ (Figs. 4e and 4g). Amongst these 15 genes (Fig. 4e), *TM4SF4* and *HNF4A* were identified in the triple overlap in the transcriptomics data for *HNF1A*^*+/H126D*^ endocrine progenitors, *HNF1A*^*+/T260M*^ islets and *HNF1A*^*+/−*^ hESC-derived β cells (Fig. 3e).

Collectively, our RNA-Seq and ChIP-Seq results suggested that the heterozygous HNF1A H126D mutation is associated with reduced binding of HNF1A to specific targets, resulting in decreased expression of these genes. In the mutant endocrine progenitors, many of the downregulated genes are involved in β cell survival and function, which may account for the deficient insulin secretion observed in our MODY3 patients.

### Reduced expression of genes involved in insulin secretion

**Fig. 5:**
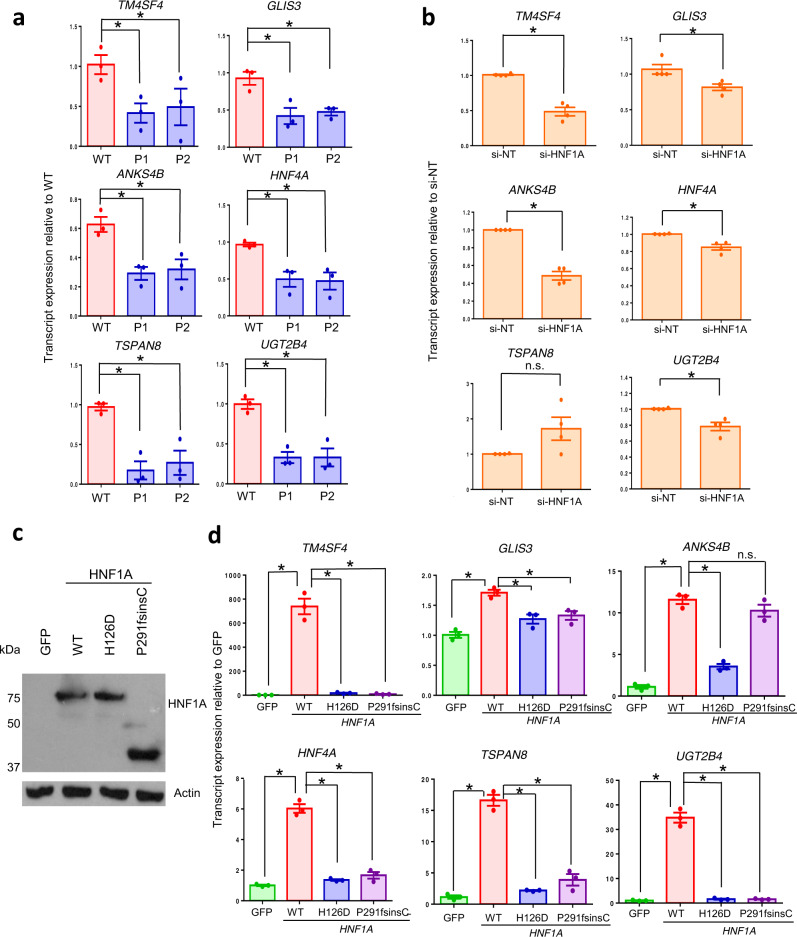
*HNF1A*^*+/H126D*^ mutation reduces the expression of genes involved in pancreas development, β cell survival and insulin secretion. **a** RT-qPCR analysis of *TM4SF4*, *GLIS3*, *ANKS4B*, *HNF4A*, *TSPAN8* and *UGT2B4* transcripts in WT (red) and mutant (blue) hPSC-derived endocrine progenitors. *n* = 3 independent experiments. *P*-value for *TM4SF4* = 0.0238 (P1), 0.0390 (P2); *GLIS3* = 0.0239 (P1), 0.0184 (P2); *ANKS4B* = 0.0081 (P1), 0.0260 (P2); *HNF4A* = 0.0385 (P1), 0.0449 (P2); *TSPAN8* = 0.0112 (P1), 0.0355 (P2); *UGT2B4* = 0.0020 (P1), 0.0130 (P2). **b** RT-qPCR analysis of EndoC-βH1 cells transfected with *HNF1A* siRNA (si-HNF1A) and non-targeting siRNA as negative control (si-NT). *n* = 4 independent experiments. *P*-value for *TM4SF4* = 0.0029; *GLIS3* = 0.0240; *ANKS4B* = 0.0017; *HNF4A* = 0.0172; *UGT2B4* = 0.0226. **c** Western blot analysis for HNF1A protein in AD-293 cells overexpressed with GFP and various *HNF1A* constructs. *n* = 3 independent experiments. **d** RT-qPCR analysis of *TM4SF4*, *GLIS3*, *ANKS4B*, *HNF4A*, *TSPAN8* and *UGT2B4* transcripts in AD-293 cells overexpressed with GFP (green) and various WT (red), H126D (blue) and P291fsinsC (purple) *HNF1A* constructs. *n* = 3 independent experiments. *P*-value for *TM4SF4* = 0.0076 (WT), 0.0078 (H126D), 0.0077 (P291fsinsC); *GLIS3* = 0.0005 (WT), 0.0121 (H126D), 0.0171 (P291fsinsC); *ANKS4B* = 0.0005 (WT), 0.0004 (H126D); *HNF4A*: 0.0026 (WT), 0.0078 (H126D), 0.0886 (P291fsinsC); *TSPAN8* = 0.0015 (WT), 0.0034 (H126D), 0.0005 (P291fsinsC); *UGT2B4* = 0.0035 (WT), 0.0034 (H126D), 0.0035 (P291fsinsC). WT wild-type, P1 patient 1, P2 patient 2. GFP green fluorescent protein. RT-qPCR quantitative reverse transcription polymerase chain reaction. hPSC human pluripotent stem cells. For all statistical analysis: Error bars represent standard error of mean (SEM). Unpaired one-tailed Student’s *t*-test was performed. Asterisk indicates *P*-value < 0.05. n.s. non-significant. “See also Fig. S7.” Source data are provided as a Source data file.

Subsequently, we proceeded to validate 13 out of 15 of these direct targets of HNF1A downregulated in *HNF1A*^*+/H126D*^ mutant pancreatic endocrine progenitors (Figs. 4e, 5a and S7a; primers could not be designed for two genes). While RT-qPCR analyses did not reveal any change in *HNF1A* transcript expression between WT and mutant cells (Fig. S7a), we found *TM4SF4, GLIS3, ANKS4B, HNF4A, TSPAN8* and *UGT2B4* transcripts to be downregulated in mutant *HNF1A*^*+/H126D*^ endocrine progenitors from both P1 and P2 as compared to WT cells (Fig. 5a).

To confirm that these genes are directly regulated by HNF1A in human β cells, we knocked down *HNF1A* in the EndoC-βH1 human β cell line. Knockdown of *HNF1A* transcript resulted in a significant decrease in the transcript expression of *TM4SF4, GLIS3, ANKS4B, HNF4A, UGT2B4*, *GPR39, PDGFA, FGFR4* and *LAMA1* (Figs. 5b and S7b). We also overexpressed WT and various mutant *HNF1A* constructs in the AD-293 human kidney cell line to evaluate their gene regulatory function on these putative HNF1A target genes (Figs. 5c, 5d, S7c and S7d). AD-293 cells have a low endogenous expression of HNF1A, making it a clean system to study the effects of *HNF1A* overexpression. P291fsinsC, being the most common MODY3 mutation, was used as a negative control. While both WT and mutant HNF1A transcripts and proteins were overexpressed at similar levels (Figs. 5c and S7c), WT *HNF1A* overexpression significantly increased the transcript expression levels of *TM4SF4, GLIS3, HNF4A, TSPAN8*, *UGT2B4*, *GPR39* and *FGFR4*, while mutant H126D or P291fsinsC HNF1A overexpression showed significantly diminished increase compared to WT (Figs. 5d and S7d). Together, these results demonstrated that HNF1A protein regulates several genes that are involved in pancreas development, β cell survival, insulin secretion and association with T2D. In particular, *TM4SF4* and *HNF4A* consistently appeared in the various RNA-Seq and ChIP-Seq datasets (Figs. 3e and 4e), indicating that they could be important targets of HNF1A protein. MODY3 mutations, such as *HNF1A* H126D or P291fsinsC, disrupt the expression of these genes, which may contribute to the defective β cell phenotype in MODY3 patients.

### No defects in calcium signaling nor potassium channels

**Fig. 6:**
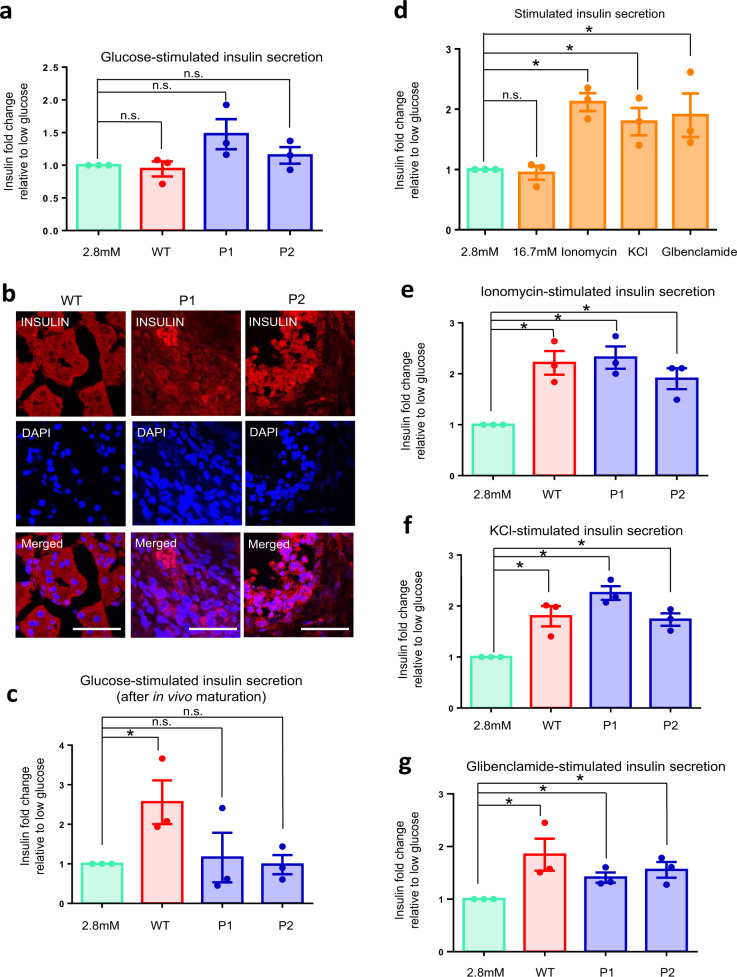
MODY3-hiPSC-derived mutant β-like cells did not exhibit defects in calcium signaling nor potassium channels. **a** Glucose-stimulated insulin secretion of WT (red) and patient-specific (blue) hPSC-derived β-like cells in vitro. *n* = 3 independent experiments. **b** Immunohistochemistry stain for INS (red) and nuclear stain using DAPI (blue) in β-like cells after in vivo maturation in mouse kidney capsule for 23 weeks. *n* = 3 independent experiments. (scale bar: 50 μm). **c** Glucose-stimulated insulin secretion of WT (red) and patient-specific hPSC-derived β-like (blue) cells after in vivo maturation in mouse kidney capsule for 23 weeks. *n* = 3 independent experiments; *p* = 0.0478 (WT). **d** Insulin secretion of WT β-like cells after stimulation with 2.8 mM glucose (green), 16.7 mM glucose, 10 μM ionomycin, 30 mM KCl or 100 μM glibenclamide. *n* = 3 independent experiments; *p* = 0.0017 (ionomycin), 0.0245 (KCl), 0.0672 (glibenclamide). **e** Ionomycin-stimulated insulin secretion of WT (red) and patient-specific (blue) hPSC-derived β-like cells. *n* = 3 independent experiments; *p* = 0.0064 (WT), 0.0038 (P1), 0.0117 (P2). or **f** KCl-stimulated insulin secretion of WT (red) and patient-specific (blue) hPSC-derived β-like cells. *n* = 3 independent experiments; *p* = 0.0158 (WT), 0.0007 (P1), 0.0037 (P2). **g** Glibenclamide-stimulated insulin secretion of WT (red) and patient-specific (blue) hPSC-derived β-like cells. *n* = 3 independent experiments; *p* = 0.0496 (WT), 0.0136 (P1), 0.0203 (P2). All insulin fold changes are normalized to insulin amounts secreted at 2.8 mM glucose (green) under each condition. WT wild-type, P1 patient 1, P2 patient 2. hPSC human pluripotent stem cells. For all statistical analysis: Error bars represent standard error of mean (SEM). Unpaired one-tailed Student’s *t*-test was performed. Aterisk indicates *P*-value < 0.05. “See also Fig. S8.” Source data are provided as a Source data file.

MODY3 patients are characterized by defective insulin secretion. Next, to investigate the effect(s) of gene dysregulation caused by the *HNF1A*^*+/H126D*^ mutation on pancreatic β cell function, we differentiated the two WT and six mutant hPSC lines into pancreatic β-like cells and evaluated their insulin secretion function. High glucose challenge first revealed that these hPSC-derived β-like cells were not functional in vitro, with no significant increase in insulin secretion (<2-fold insulin secretion; Fig. 6a). We then transplanted these β-like cells into the kidney capsule of mice for functional maturation over 23 weeks. After 23 weeks, we harvested the transplanted cells and performed immunohistochemistry staining to confirm the presence of INS^+^ β-like cells (Fig. 6b). After in vivo maturation, the WT β-like cells secreted insulin (≥2-fold insulin fold change) and C-peptide (≥1.5-fold C-peptide fold change) in response to high glucose, while the mutant *HNF1A*^*+/H126D*^ β-like cells were non-responsive (Figs. 6c and S8a), consistent with the pathophysiology of our MODY3 patients. This data indicated that our hPSC-derived β cell model is relevant and appropriate for MODY3 disease modeling studies.

In the stimulus-secretion coupling of β cells, the entry of glucose into the β cell via glucose transporters GLUT1, GLUT2 and/or GLUT3^39^ triggers the onset of glycolysis which increases ATP production^40^ (Fig. S8b). This initiates the closure of ATP-sensitive K^+^ channels which then depolarizes the β cell membrane^41^, resulting in the opening of voltage-gated Ca^2+^ channels. This allows Ca^2+^ entry into the cell, which triggers the fusion of insulin granules with the β cell membrane, and the eventual exocytosis of insulin^42,43^.

To investigate the individual component(s) of the stimulus-secretion coupling pathway that may possibly be affected in MODY3 mutant β-like cells, we challenged the β-like cells with various chemical secretagogues targeting the voltage-gated Ca^2+^ or K_ATP_ channels (Fig. S8b). Ionomycin, a calcium ionophore, increases the intracellular Ca^2+^ concentration, bypassing the physiological regulation of voltage-gated Ca^2+^ channels. KCl increases the intracellular K^+^ concentration, hence bypassing the regulation of the K_ATP_ channels. Glibenclamide, a glucose-lowering sulfonylurea, binds and closes the K_ATP_ channels, hence depolarizing the β cell membrane. Our in vitro WT hPSC-derived β-like cells had functional voltage-gated Ca^2+^ and K_ATP_ channels as demonstrated by their ability to result in 2-fold insulin secretion (as is typical of human β cells) upon 10 μM ionomycin, 30 mM KCl or 100 μM glibenclamide stimulation (Fig. 6d).

When *HNF1A*^*+/H126D*^ mutant β-like cells were compared with WT β-like cells upon ionomycin-, KCl- or glibenclamide-stimulated insulin secretion, no significant differences were observed (Figs. 6e–g), suggesting that MODY3 mutant β-like cells had functional voltage-gated Ca^2+^ and K_ATP_ channel activities. These data suggested that the mechanism for defective glucose-stimulated insulin secretion in MODY3 mutant β cells is upstream of K_ATP_ channel signaling.

### Decreased GLUT2, glucose uptake and ATP production

**Fig. 7:**
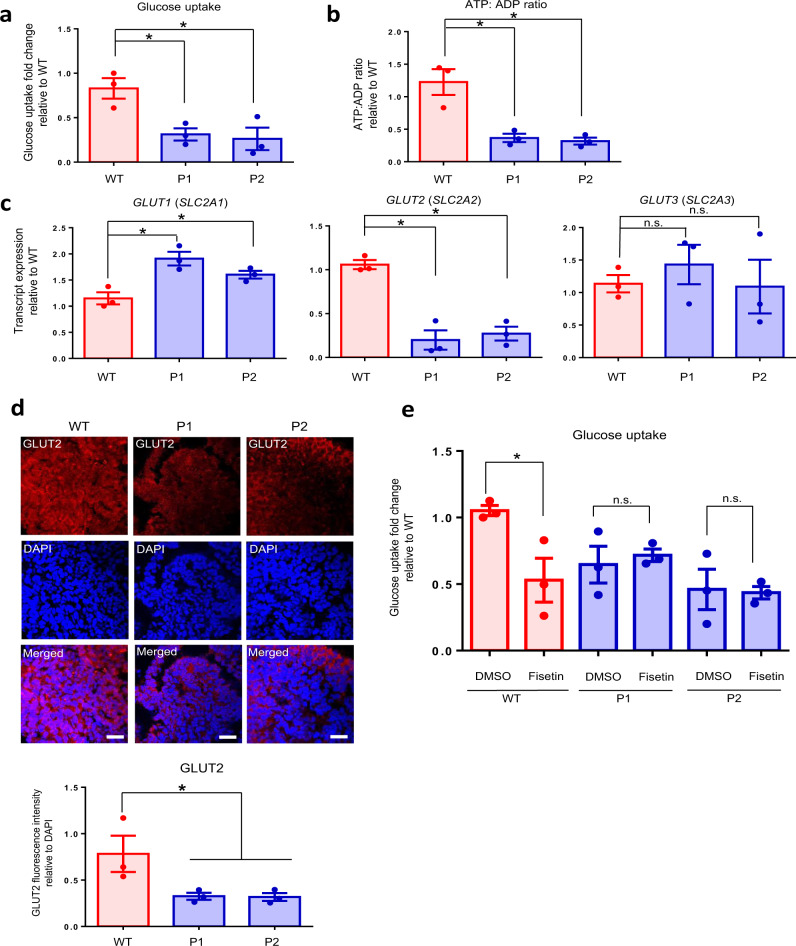
Decreased GLUT2 expression, glucose uptake and ATP production in MODY3 mutant β-like cells. **a** Glucose uptake in WT (red) and patient-specific (blue) hPSC-derived β-like cells. *n* = 3 independent experiments; *p* = 0.0182 (P1), 0.0294 (P2). **b** ATP: ADP ratio in WT (red) and patient-specific (blue) hPSC-derived β-like cells. *n* = 3 independent experiments; *p* = 0.0144 (P1), 0.0113 (P2). **c** RT-qPCR analysis of *GLUT1, GLUT2* and *GLUT3* transcripts in WT (red) and mutant (blue) hPSC-derived endocrine progenitors. *n* = 3 independent experiments; *P*-value for *GLUT1* = 0.0125 (P1), 0.0373 (P2); *GLUT1* = 0.0068 (P1), 0.0021 (P2). **d** Immunohistochemistry stain of GLUT2 (red) and DAPI (blue) in WT and mutant differentiated day 20 endocrine progenitors. *n* = 3 independent experiments. (scale bar:100 μm). **e** Glucose uptake in WT (red) and patient-specific (blue) hPSC-derived β-like cells in the presence of dimethyl sulfoxide (DMSO) or 60 μM GLUT2 inhibitor, fisetin. Glucose uptake fold changes are normalized to glucose uptake amount in the presence of DMSO in WT. *n* = 3 independent experiments; *p* = 0.0362 (WT). WT wild-type, P1 patient 1, P2 patient 2. hPSC human pluripotent stem cells. For all statistical analysis: Error bars represent standard error of mean (SEM). Unpaired one-tailed Student’s *t*-test was performed. Asterisk indicates *P*-value < 0.05. n.s. non-significant. “See also Figs. S9 and S10.” Source data are provided as a Source data file.

To determine if the *HNF1A*^*+/H126D*^ mutation had any effects on glucose uptake into the β-like cells, we compared the glucose uptake between WT and mutant β-like cells. Interestingly, the glucose uptake in *HNF1A*^*+/H126D*^ mutant β-like cells from both MODY3 patients was significantly lower than that in the WT β-like cells (Fig. 7a). To investigate if the reduced glucose uptake affected energy production in the mutant β-like cells, we compared the ADP to ATP conversion between WT and mutant β-like cells. Indeed, the ATP:ADP ratio in *HNF1A*^*+/H126D*^ mutant β-like cells from both MODY3 patients was significantly reduced as compared to that in the WT β-like cells (Fig. 7b). These data suggested that decreased glucose uptake can reduce the ATP production and may contribute to decreased insulin secretion in MODY3 patients.

Next, we investigated the cause of the reduced glucose uptake in the *HNF1A*^*+/H126D*^ β-like cells. We evaluated the gene expression profile of human β cell glucose transporters *GLUT1* (*SLC2A1*), *GLUT2* (*SLC2A2*) and *GLUT3* (*SLC2A3*) in both WT and *HNF1A*^*+/H126D*^ mutant endocrine progenitors. Intriguingly, *GLUT2* transcripts were significantly downregulated, accompanied by a slight increase in *GLUT1* transcript expression in mutants as compared to WT cells (Fig. 7c). No difference in *GLUT3* expression was observed. This finding is surprising since most existing literature have reported GLUT1 and GLUT3, rather than GLUT2, as the principal glucose transporters in human pancreatic β cells^44^. Immunostaining analyses further confirmed the decrease in GLUT2 protein expression in mutant pancreatic cells (Fig. 7d). We further confirmed the co-expression of HNF1A and GLUT2 with INS in the INS + β-like cells (Fig. S9).

To ascertain the function of GLUT2 in glucose uptake in human β cells, we used a GLUT2 inhibitor, fisetin^45^, to block GLUT2 function in the hPSC-derived pancreatic β-like cells. We treated the β-like cells with increasing dosages of 0, 30, 60, 90 and 120 μM fisetin to determine the specificity of the GLUT2 inhibitor (Figs. S10a and S10b). The increasing fisetin concentration reduced the glucose uptake in the WT β-like cells in a dosage-dependent manner (Pearson’s R^2^ ~0.8), and reached maximum effect at 60 μM. However, the increasing fisetin dosage had no effect on the glucose uptake in the mutant P1 and P2 β-like cells (Pearson’s R^2^ ~0.05 to ~0.2). We found that 60 μM fisetin reduced glucose uptake in WT β-like cells to similar levels as those in MODY3 mutant β-like cells (Fig. 7e). Exposure to fisetin demonstrated no further effect on the glucose uptake ability in the mutant β-like cells, indicating that the loss of glucose uptake capacity caused by reduced GLUT2 expression as a result of the *HNF1A*^*+/H126D*^ mutation had possibly reached maximal effect. Therefore, our results indicate that the downregulated GLUT2 expression in our MODY3 patient hiPSC-derived pancreatic cells is associated with reduced glucose uptake, reduced ATP:ADP ratio and reduced GSIS.

### Reduced ability of HNF1A to upregulate *GLUT2* gene expression

**Fig. 8:**
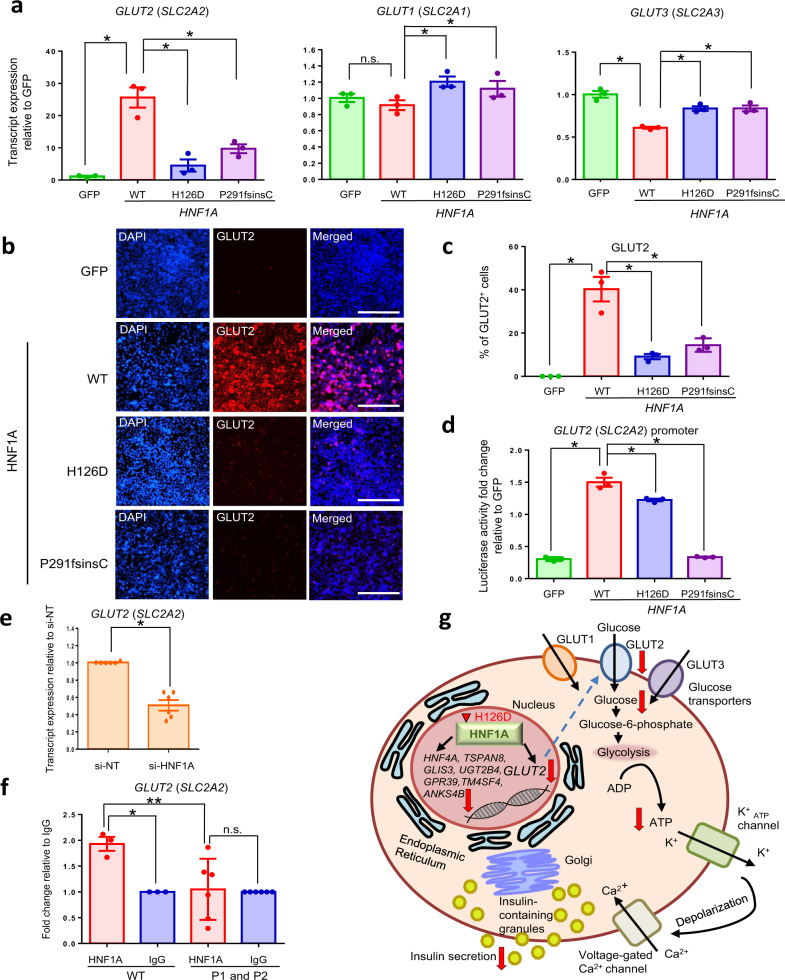
HNF1A H126D mutation reduced the ability of HNF1A to upregulate *GLUT2* gene expression. **a** RT-qPCR analysis of *GLUT2*, *GLUT1* and *GLUT3* transcripts in AD-293 cells overexpressed with GFP (green) and various WT (red), H126D (blue) and P291fsinsC (purple) *HNF1A* constructs. *n* = 3 independent experiments; *P*-value for *GLUT2* = 0.0156 (WT), 0.0080 (H126D), 0.0227 (P291fsinsC); *GLUT1* = 0.0296 (H126D); *GLUT3* = 0.006 (WT), 0.0063 (H126D), 0.0141 (P291fsinsC). **b** Immunohistochemistry stain for GLUT2 (red) and nuclear stain using DAPI (blue) in AD-293 cells overexpressed with GFP and various *HNF1A* constructs. *n* = 3 independent experiments. (scale bar:100 μm). **c** Immunofluorescence data was quantified by cell counting using ImageJ across three representative images per condition in AD-293 cells overexpressed with GFP (green) and various WT (red), H126D (blue) and P291fsinsC (purple) *HNF1A* constructs. **d** Luciferase reporter analysis of *GLUT2* promoter activity in AD-293 cells overexpressed with GFP (green) and various WT (red), H126D (blue) and P291fsinsC (purple) *HNF1A* constructs (*n* = 3). **e** RT-qPCR analysis of *GLUT2* transcripts in EndoC-βH1 cells transfected with *HNF1A* siRNA (si-HNF1A) and non-targeting siRNA as negative control (si-NT). *n* = 6 independent experiments. *p* = 0.0012. **f** ChIP-qPCR analysis of HNF1A binding onto *GLUT2* promoter in hPSC-derived endocrine progenitors, using HNF1A antibody (red) and negative control IgG (blue). *n* = 3 independent experiments. One-way ANOVA was performed for group comparison (*p* = 0.0132, F-critical = 6.3419). Tukey post-hoc test was done for pairwise comparison, *p = 0.0209, **p = 0.0152. **g** Summary diagram showing the components of stimulus-secretion coupling in human β cell affected by HNF1A H126D mutation. WT wild-type, P1 patient 1, P2 patient 2. GFP green fluorescent protein. ChIP-Seq Chromatin immunoprecipitation sequencing. RT-qPCR quantitative reverse transcription polymerase chain reaction. hPSC human pluripotent stem cells. For all statistical analysis: Error bars represent standard error of mean (SEM). Unpaired one-tailed Student’s *t*-test was performed for all statistical analysis, otherwise stated. Asterisk indicates *P*-value < 0.05. n.s. non-significant. “See also Figs. S7 and S11.” Source data are provided as a Source data file.

To conclusively demonstrate a direct relationship between HNF1A and human *GLUT2* gene expression, we overexpressed WT and various mutant *HNF1A* constructs in AD-293 human kidney cell line (Fig. S7d) and evaluated their effects on *GLUT2* gene expression. WT *HNF1A* overexpression significantly increased the transcript and protein expression level of GLUT2, while H126D or P291fsinsC overexpression increased the GLUT2 expression to a significantly lesser extent compared to WT (Figs. 8a–c). Luciferase assays performed on a *GLUT2* promoter containing HNF1A-binding sites^46^ demonstrated that HNF1A H126D and P291fsinsC were less effective in increasing *GLUT2* promoter transcriptional activity as compared to WT HNF1A (Fig. 8d). Knockdown of *HNF1A* in EndoC-βH1 human β cell line (Fig. S7b) also resulted in a significant decrease in *GLUT2* transcript expression but not that of *GLUT1* or *GLUT3* transcripts (Figs. 8e and S11a). Finally, ChIP-Seq analyses confirmed the loss of binding in the *GLUT2* promoter in the mutant endocrine progenitors. We observed a reduced peak on a known HNF1A-binding site^46^ in the mutants compared to the WT, albeit not a statistically significant one (Fig. S11b). This loss of binding was supported by our ChIP-qPCR, which showed that HNF1A is directly bound onto *GLUT2* promoter in WT but not in mutant hPSC-derived endocrine progenitors (Fig. 8f), hence confirming this direct gene regulation relationship. Together, our results reinforced the importance of HNF1A in directly regulating *GLUT2* gene and protein expression in human β cells, and established that the HNF1A H126D mutation reduces the expression of GLUT2 (Fig. 8g). A similar lack of GLUT2 expression observed in the *HNF1A* P291fsinsC mutant suggests that impaired glucose uptake capacity may be common amongst diabetes patients whose β cells harbor various *HNF1A* gene mutations.

Overall, our results suggested that human HNF1A is responsible for the active regulation of GLUT2 expression and, heterozygous *HNF1A* mutation can perturb GLUT2 expression to severely impair glucose uptake and disrupt ATP production in human β cells (Fig. 8h). This reduced glucose uptake and ATP production could have contributed to the decreased insulin secretion observed in mutant β-like cells and in MODY3 patients.

## Discussion

Here, we leveraged upon MODY3 patient-derived hiPSCs to reveal the impact of a patient-specific heterozygous *HNF1A*^*+/H126D*^ mutation on essential glucose transporter GLUT2 expression and glucose uptake function in pancreatic β cells, thereby affecting the insulin secretory function of pancreatic β cells in humans.

First, our computational modeling experiments predicted that the H126D mutation in the DNA-binding domain of HNF1A caused a loss of hydrogen bonds between HNF1A protein and target DNA complexes. The mutation is predicted to alter interactions between the mutated residue and its neighboring residues, including K205 which is known to interact directly with DNA^12^ and is also a site known to be SUMOylated by E3 SUMO ligase PIASγ^47^. The hydrogen bond formed between the side chains of K205 and N127 has been suggested to maintain a stable interface in the POU_S_ and POU_H_ domains of HNF1A protein^12^. Hence, K205Q, a known MODY3 mutation^48^, is predicted to have altered POU_S_-POU_H_ domain interactions, leading to reduced transcriptional activity^12^. The altered interaction with K205 in the H126D mutant may similarly destabilize the POU_S_-POU_H_ domain interactions, disrupting the transcriptional activity of HNF1A. The loss of flexibility in the mutant HNF1A protein compared to WT protein could also hinder induced-fit binding to DNA which is necessary for optimal transcriptional activation^49^.

We found 1682 genes to be significantly downregulated in the MODY3 endocrine progenitors, many of which are involved in transport, localization, signaling and secretion. In contrast, only 512 genes were upregulated in the mutants, suggesting that the H126D mutation caused decreased binding between the mutant HNF1A protein and its target genes, resulting in a dysregulation of downstream gene expression. This is further supported by the reduced promoter-binding activity of HNF1A protein in the MODY3 endocrine progenitors as compared to WT cells, evident from our ChIP-Seq analyses.

Among the genes that were significantly downregulated in our MODY3 *HNF1A*^*+/H126D*^ endocrine progenitors, 32 of them were also found to have a loss of binding by the mutant HNF1A protein, hence suggesting that the expression of these genes was downregulated due to a loss of binding by the mutant HNF1A. Several of these genes, including *TM4SF4, GLIS3*, *ANKS4B, GPR39, HNF4A, FGFR4, TSPAN8* and *UGT2B4*, are involved in pancreas development and function. WT HNF1A has been reported to regulate *TM4SF4* expression in human pancreas^50^ and *Tm4sf4* expression is reduced in *Hnf1a*^−/−^ mouse islets^51^. *GLIS3* expression is reduced in *HNF1A*^*+/−*^ hPSC-derived β-like cells^10^. GLIS3 mutations are known to cause neonatal diabetes^52^ and *GLIS3* gene polymorphisms are also associated with MODY, while *GLIS3* variants are strongly associated with T1D and T2D in several populations^21,53–55^. *Anks4b* transcription is also found to be synergistically activated by HNF4A and HNF1A in HEK293 cells, and disruption of the HNF1A-binding site in the *Anks4b* promoter suppressed the synergistic activation of *Anks4b* promoter activity^22^. The overexpression of *FGFR4* in pancreatic cancer is found to be mediated by an intronic enhancer that is activated by HNF1A, and mutations in the HNF1A-binding site reduced the enhancer activity^56^. *Fgfr4*^*−/−*^ knockout mice exhibited glucose intolerance and insulin resistance, while *FGFR4* polymorphisms can increase insulin secretion in mice pancreatic islets^33,57^. Similarly, the *GPR39* promoter is shown to be bound by HNF1A in human hepatocytes^58^. Disruption of the HNF1A-binding site in *GPR39* promoter significantly impaired the transcriptional activity of the *GPR39* promoter^59^, suggesting that HNF1A is involved in the regulation of *GPR39* gene expression. GPR39 is required for insulin secretion, and *Gpr39*^*−*/*−*^ mice showed increased glucose levels, decreased serum insulin levels and impaired glucose tolerance^24,25^. HNF1A is observed to bind the *HNF4A* promoter in mice and human pancreatic cells^60,61^ and *Hnf4a* expression is reduced in *Hnf1a*^*−*/*−*^ mice^60^. *HNF4A* encodes a transcription factor that is essential for glucose-stimulated insulin secretion in mouse pancreatic β cells, and *HNF4A* mutations are known to cause MODY1^26,27^. Importantly, *TM4SF4* and *HNF4A* are also downregulated in *HNF1A*^*+/T260M*^ islets^9^ and in *HNF1A*^*+/−*^ hESC-derived β-like cells^10^, indicating that these two genes are commonly perturbed in MODY3.

To the best of our knowledge, *TSPAN8* and *UGT2B4* have not been previously identified as targets bound by HNF1A. *TSPAN8* variants have been associated with T2D^35,36^, while Rifampin, a UGT2B4 enzyme inducer, is currently administered as a drug to manage T2D^38^. Rodent studies reported that *Tspan8*^*−/−*^ knockout mice exhibited normal fasting insulin and glucose levels, as well as normal insulin sensitivity^62^. TSPAN8 expression is nearly absent in mice pancreas, while TSPAN8 is abundantly expressed in the human pancreas^62^. The lack of *Tspan8* in mouse pancreas hence explains why previous rodent β cell studies did not identify *Tspan8* as a target of HNF1A nor its association with MODY3, and highlights the significance of studying MODY3 in human pancreatic cells. The lack of binding and reduced transcription of *TSPAN8* and *UGT2B4* in the *HNF1A*^*+/H126D*^ endocrine progenitors, and their lowered expression in AD-293 cells overexpressed with *HNF1A* H126D or P291fsinsC show that *HNF1A* mutations dysregulate these genes. Further studies are required to investigate their roles in MODY3. Overall, our RNA-Seq and ChIP-Seq results indicate that *HNF1A* mutations alter the expression of several genes that are involved in pancreas development, β cell survival and insulin secretion, which may collectively contribute to the pathogenesis of MODY3.

MODY3 patients are clinically linked with defective insulin secretion. Our systematic stepwise evaluation of the stimulus-secretion coupling pathway did not reveal significant defects in the voltage-gated Ca^2+^ and K_ATP_ channel activities. Calcium ionophore, KCl, and low-dose sulfonylurea (glibenclamide) were all able to effectively stimulate insulin secretion in MODY3 mutant β-like cells comparable to that of WT β-like cells. This indicated that the heterozygous *HNF1A*^*+/H126D*^ mutation affected the stimulus-secretion coupling pathway upstream of the K_ATP_ channels in the β cells.

Next, we evaluated glucose entry into the β cells. Interestingly, heterozygous *HNF1A*^*+/H126D*^ mutation only resulted in a decrease in *GLUT2* but not that of *GLUT1* or *GLUT3* expression in mutant β-like cells. This was associated with reduced glucose uptake capacity in the MODY3 mutant β-like cells. Potent non-competitive GLUT2 inhibitor fisetin^45,63^ effectively reduced glucose uptake in WT β-like cells but not in mutant β-like cells, likely due to the already low GLUT2 levels in the mutants. We then provided evidence that HNF1A is directly bound onto *GLUT2* promoter and that our patient-specific *HNF1A* H126D mutation failed to upregulate *GLUT2* promoter activity effectively as compared to WT HNF1A^46^, resulting in decreased *GLUT2* transcript and protein expression. Similarly, we also found downregulation of *GLUT2* in both *HNF1A*^*+/T260M*^ islets^9^ and *HNF1A*^*+/−*^ hESC-derived β-like cells^10^. These collective data confirmed the clear and direct effects of a heterozygous *HNF1A*^*+/H126D*^ mutation that led to decreased GLUT2 expression and function, possibly accounting for the defective insulin secretion in MODY3 human β cells.

The implication of GLUT2 in MODY3 in our work sheds light on the role of this glucose transporter in human β cells. In mouse β cells, GLUT2 is established as the predominant glucose transporter and is known to be involved in insulin secretion. *Glut2*^*−*/*−*^ mice are hyperglycemic, hypoinsulinemic and die from lethal neonatal diabetes. *Glut2*^*−*/*−*^ mouse islets exhibit reduced glucose uptake and impaired GSIS, while the re-expression of GLUT2 in the islets restores normal GSIS^64,65^. However, while the role of GLUT2 in mouse β cells has been well-established, the role of GLUT2 in human β cells has remained controversial. Unlike in rodent β cells where *Glut2* is the predominantly expressed glucose transporter with expression more than tenfold higher than *Glut1*, human β cells predominantly express *GLUT1* and *GLUT3*, with 2.8 and 2.7 fold higher expression than *GLUT2*, respectively^44^. In addition, human β cells have 100-fold lower GLUT2 abundance than rat β cells, which correlated to ten times lower glucose uptake in human β cells than rat β cells^39^. Hence, the lower abundance of GLUT2 in human β cells suggested that it may not be the principal glucose transporter in human β cells and is probably not an essential glucose sensor of human β cells^39,44^.

Yet, despite the purported irrelevance of GLUT2 in human β cells, homozygous *GLUT2* mutations can cause Fanconi–Bickel syndrome and neonatal diabetes^66^, and SNPs in *GLUT2* are associated with the conversion from impaired glucose tolerance to T2D^67^. Similarly, *GLUT2* variants are also associated with impaired fasting glucose and diabetes^54,68,69^. Furthermore, Fanconi–Bickel syndrome-associated mutations in *GLUT2* were found to decrease glucose transport capacity by human GLUT2 in *Xenopus* oocytes^70^. Hence, the association of *GLUT2* mutations in Fanconi–Bickel syndrome and diabetes pathology supports an imperative role played by GLUT2 in human β cells despite its lower abundance^71^. By demonstrating that a patient-specific heterozygous *HNF1A*^*+/H126D*^ mutation causes decreased GLUT2 expression in human β cells, correlated with a reduction in glucose uptake and ATP production, we posit that the defective insulin secretion in MODY3 β cells is at least partly contributed by the lowered availability of glucose in these β cells. Our study implicates the involvement of GLUT2 in the pathogenesis of MODY3 in humans and further supports the importance of GLUT2 in human β cell function. Furthermore, we found decreased *GLUT2* expression in *HNF1A*^*+/T260M*^ islets^9^, and demonstrated that the most common MODY3 mutation P291fsinsC also failed to increase GLUT2 expression, suggesting that reduced GLUT2-facilitated glucose uptake may be common in most MODY3 β cells. Better facilitation of glucose entry into these β cells may be a simple means to restore insulin secretion function in MODY3.

In addition to MODY3, *HNF1A* variants have also been implicated in T2D^72–74^. Our study supports the role of *HNF1A* in T2D given that the *HNF1A* H126D mutation resulted in a loss of binding and downregulation of various genes (*TSPAN8, LAMA1* and *UGT2B4*) associated with T2D. Hence, our findings offer insights into the involvement of *HNF1A* in T2D.

In conclusion, our study has successfully established an in vitro platform for MODY3 disease modeling via the differentiation of MODY3 patient-derived hiPSCs into pancreatic β-like cells. This model has allowed us to elucidate the molecular pathways that are involved in the pathology behind MODY3 disease, and identified a list of genes including *GLUT2* that are potentially implicated in the disease. Importantly, our results showed that *HNF1A* mutations cause decreased GLUT2 expression, which is associated with reduced glucose uptake and ATP production, and hence likely contributes to the lack of glucose-stimulated insulin secretion in MODY3 patients.

## Methods

### Multiple sequence alignment

To identify the conservation of HNF1A protein, a HomoloGene^75^ search was performed using the keyword “Homo sapiens HNF1A”. Additionally, we compared the isoform sequence amongst various species using MUSCLE (version 3.6)^76^ and visually inspected the similarity or difference of the sequence in multiple sequence alignment view.

### Molecular dynamics (MD) simulations

#### Preparation of structures

The crystal structure of HNF1A bound to DNA (PDB code 1IC8)^12^ was used as the starting structure for molecular dynamics (MD) simulations. Unresolved HNF1A residues (85–86, 181–200, 277–278 in chain A and 180–200 in chain B) were modelled using the ModLoop web server^77^ while the H126D mutation was introduced into both HNF1A chains using PyMOL^78^. The N- and C- termini of HNF1A were capped by acetyl and N-methyl groups, respectively. A total of four systems were set up: wild-type (WT) HNF1A bound to DNA, H126D mutant HNF1A bound to DNA, monomeric WT HNF1A, and monomeric H126D mutant HNF1A. Chain B from the crystal structure was used for the monomeric HNF1A simulations. The software package PDB2PQR^79^ was used to determine the protonation states of residues. Each system was then solvated with TIP3P water molecules^80^ in a periodic truncated octahedron box such that its walls were at least 10 Å away from the complex or protein, followed by charge neutralization with sodium ions.

#### Conventional molecular dynamics (cMD) simulations

Energy minimizations and MD simulations were performed with the PMEMD module of AMBER 14^81^ using the ff14SB^82^ force field, which includes the ff99bsc0 corrections for DNA^83^. All bonds involving hydrogen atoms were constrained by the SHAKE algorithm^84^, allowing for a time step of 2 fs. Nonbonded interactions were truncated at 9 Å, while the particle mesh Ewald method^85^ was used to account for long-range electrostatic interactions under periodic boundary conditions. Weak harmonic positional restraints with a force constant of 2.0 kcal mol^*−*1^ Å^*−*2^ were placed on the protein and DNA non-hydrogen atoms during the minimization and equilibration steps. Energy minimization was carried out using the steepest descent algorithm for 500 steps, followed by the conjugate gradient algorithm for another 500 steps. The systems were then heated gradually to 300 K over 50 ps at constant volume before equilibration at a constant pressure of 1 atm for another 50 ps. Finally, unrestrained equilibration (2 ns) and production (200 ns) runs were carried out at 300 K using a Langevin thermostat^86^ with a collision frequency of 2 ps^*−*1^, and 1 atm using a Berendsen barostat^87^ with a pressure relaxation time of 2 ps. Five independent MD simulations were carried out on each of the systems. Trajectory movies were generated using VMD^88^.

#### Accelerated molecular dynamics (aMD) simulations

Dual-boost aMD simulations, in which both dihedral (dih) energy and total potential energy (PE) are boosted, were initiated from the final structure of the corresponding cMD simulation runs. The average of the energies obtained from the five cMD simulations of the H126D mutant HNF1A–DNA complex were used to calculate the boost parameters for aMD. The following boost parameters were used:

$${\alpha }_{{\rm{PE}}}=0.16\times {{\rm{N}}}_{{\rm{atoms}}}=11426\,{\rm{kcal}}\,{{\rm{mol}}}^{-1}$$

$${{\rm{E}}}_{{\rm{PE}}}={{\rm{V}}}_{{\rm{PE}}\_{\rm{avg}}}+0.16\times {{\rm{N}}}_{{\rm{atoms}}}=-217953\,{\rm{kcal}}\,{{\rm{mol}}}^{-1}$$

$${\alpha }_{{\rm{dih}}}=4/5\times {{\rm{N}}}_{{\rm{res}}}=347\,{\rm{kcal}}\,{{\rm{mol}}}^{-1}$$

$${{\rm{E}}}_{{\rm{dih}}}={{\rm{V}}}_{{\rm{dih}}\_{\rm{avg}}}+4\times {{\rm{N}}}_{{\rm{res}}}=7747\,{\rm{kcal}}\,{{\rm{mol}}}^{-1}$$

where N_atoms_ and N_res_ are the number of atoms and residues, respectively, and V_PE_avg_ and V_dih_avg_ are the average potential and dihedral energies obtained from the cMD simulations, respectively. In all, five independent 200-ns aMD simulations of the H126D mutant HNF1A–DNA complex were carried out.

#### Binding free energy calculations

Binding free energies for the HNF1A–DNA complexes were calculated using the molecular mechanics/generalized Born surface area (MM/GBSA) method^89^ implemented in AMBER 14^81^. Two hundred equally spaced snapshot structures were extracted from the last 80 ns of each of the cMD trajectories, and their molecular mechanical energies calculated with the sander module. The polar contribution to the solvation free energy was calculated by the pbsa^90^ program using the modified generalized Born (GB) model described by Onufriev et al.^91^ while the nonpolar contribution was estimated from the solvent accessible surface area using the molsurf^92^ program with γ = 0.0072 kcal Å^*−*2^ and β set to zero. Entropy changes were not computed as they have been shown to be unnecessary for ranking the binding affinities of structurally similar ligands^93^.

### Generation of hiPSCs

5 × 10^5^ human fibroblast cells were electroporated with 1 µg of plasmids, hUL, hSK and hOct4 according to the methods from Okita et al.^13^. Plasmids from Addgene: 27077-pCXLE-hOCT3/4-shp53-F; 27078-pCXLE-hSK; 27080-pCXLE-hUL. All experiments with hPSCs were approved by the NHG Domain Specific Review Board—Domain C (DSRB Approval 2013/01068), A*STAR IRB 2020–096, and all methods were performed in accordance with the Helsinki Declaration. Informed consent was obtained from all participants.

### Human PSC culture

hiPSCs generated from human fibroblast cells and H9 ESCs (WiCell Research Institute, NIHhESC-10-0062) were cultured at 37 °C with 5% CO_2_ in DMEM/F-12 supplemented with 15 mM HEPES (STEMCELL Technologies), 20% KnockOut™ serum replacement (KOSR), L-glutamine, NEAA (Life Technologies) and 10 ng/ml FGF2 (Miltenyi Biotec). hPSCs were seeded on irradiated CF-1 mouse embryonic fibroblasts (MEFs) (Gibco, A34181) and hiPSC media was replaced every 24 h. hPSCs were split when the cells reached 80% confluency. hPSCs were confirmed to be mycoplasma-free using the MycoAlert™ Mycoplasma Detection Kit (Lonza Bioscience).

### Pancreatic β cell differentiation

hiPSCs and H9 cells were differentiated along the pancreatic lineage into endocrine progenitors and β-like cells as described in ref. ^15^, with some modifications. Cells were dissociated into single cells using TrypLE^TM^ Express Enzyme and passed through 40 µm cell strainer before being re-plated at 10^6^cells/ml in mTESR medium supplemented with 10 μM Y-27632 in suspension culture on non-treated 6-well plates (Eppendorf). Cells were cultured at 37 °C with 5% CO_2_ on a rotating platform. Differentiation was initiated 48 h after cell dissociation (designated as “D0”). Three independent hiPSC lines per subject were used in each experiment, with biological triplicates analyzed for each line. Experiments were repeated at least thrice.

### Cell culture

EndoC-βH1 cells^94^ (Univercell Biosolutions) were cultured in DMEM/Low glucose (Life Technologies) supplemented with BSA (Sigma–Aldrich), penicillin/streptomycin, 2 mM L-glutamine, 50 μM 2-mercaptoethanol, 10 mM nicotinamide (Sigma–Aldrich), 5.5 μg/ml transferrin (Sigma–Aldrich) and 6.7 ng/ml sodium selenite (Sigma–Aldrich) on plates coated with 2 μg/ml fibronectin (Sigma–Aldrich) and 1% ECM (Sigma–Aldrich). AD-293 cells (Agilent, 240085) were cultured in DMEM/High glucose (Hyclone) with 10% heat-inactivated FBS (Life Technologies) and 1% NEAA (Life Technologies).

### Transfection

AD-293 cells were passaged and seeded at a cell density of 750 K cells in each well of a 6-well plate, one day prior to transfection. They were cultured in DMEM High Glucose media with GlutaMAX Supplement, Pyruvate (Invitrogen), containing 10% heat-inactivated Foetal Bovine Serum—South America (Hyclone), and 1% MEM non-essential amino acids NEAA (Invitrogen). On the day of transfection, 4 μg plasmid DNA was diluted in 250 μl of Opti-MEM® I Reduced Serum Medium (Invitrogen). Ten microlitre of Lipofectamine™ 2000 (Invitrogen) was diluted in 250 μl of Opti-MEM® I Medium and incubated for 5 min at room temperature. The diluted DNA and diluted Lipofectamine™ 2000 were then mixed and incubated for 20 min at room temperature. The DNA-Lipofectamine™ 2000 complexes were then added to each well containing AD-293 cells and 2 ml medium. The cells were incubated at 37 °C in a CO_2_ incubator for 48 h before harvest.

### Immunofluorescence staining

Cell clumps were collected at the end of the pancreatic differentiation and cryo-embedded in tissue freezing medium (Leica Biosystems). Cryo-embedded cells were sectioned and mounted onto glass slides. Sectioning was performed by the Advanced Molecular Pathology Laboratory (AMPL), A*STAR and stored at −80 °C. Cell sections or cells in monolayer culture were fixed with 4% paraformaldehyde for 20 min. For cell surface protein detection, cell sections or culture were blocked with blocking buffer (5% donkey serum or 5% bovine serum albumin in DPBS) before overnight incubation with primary antibodies at 4 °C, or 1 h in the dark at 4 °C with fluorophore-conjugated primary antibody. For intracellular protein detection, fixed cells or culture were blocked with blocking buffer containing 0.1% Triton X-100 before overnight incubation with primary antibodies at 4 °C. Following primary antibody incubation, cells were washed twice using DPBS and incubated with appropriate secondary antibody in the dark at 4 °C for 1 h. Cells were then washed at least twice using DPBS followed by nuclei staining using DAPI. Primary antibodies used were for the detection of TRA-1-60 (STEMCELL Technologies, 60064AD; 1:100), SSEA4 (STEMCELL Technologies, 60062AD; 1:100), OCT4 (Santa Cruz, sc-8628; 1:100), SOX2 (Abcam, ab97959; 1:200), NANOG (R&D Systems, AF1997; 1:100), HNF1A (Abcam, ab204306; 1:100), GLUT2 (Abcam, ab95256; 1:100. Santa Cruz, sc-9117; 1:100), and C-peptide (DSHB, GN-ID4; 1:200), PDX1 (R&D Systems, AF2419; 1:100), INS (Abcam, ab7842; 1:100). The secondary antibodies used were Alexa Fluor® 488 (Invitrogen, A11055; 1:1000), Alexa Fluor ® 488 (Invitrogen, 21202; 1:1000), Alexa Fluor® 488 (Invitrogen, A21270; 1:1000), Alexa Fluor® 488 (Invitrogen, A21206; 1:1000), Alexa Fluor® 594 (Invitrogen, A11076; 1:1000), Alexa Fluor® 594 (Invitrogen, A21203; 1:1000), Alexa Fluor® 594 (Invitrogen, A11058; 1:1000) or Alexa Fluor® 594 (Invitrogen, A21207; 1:1000). Fluorescence images were acquired with the Axiovert 200 M inverted microscope using the Axiovision LE software. Confocal images were acquired with the Olympus FV1000 inverted confocal microscope using the Olympus Fluoview v3.1 software.

### Flow cytometry

Cell clumps were dissociated into single cells using TrypLE^TM^ Express (Life Technologies) at 37 °C and passed through a 40 μm cell strainer. Single cells were fixed with 4% paraformaldehyde on ice for 30 min, then blocked in FACS buffer (5% FBS in DPBS) with (for intracellular protein) or without (for cell surface protein) 0.1% Triton X-100 on ice for 1 h, followed by incubation with primary antibodies overnight at 4 °C. The primary antibodies used were for the detection of HNF1A (Abcam, ab96777; 1:100), GATA4 (Thermo Fisher Scientific, MA5-15532; 1:100), PDX1 (Abcam, ab47308; 1:100), INS (Abcam, ab7842; 1:100) and NKX6.1 (LifeSpan BioSciences, LS‑C124275; 1:100). Cells were washed twice with FACS buffer and incubated with secondary antibodies in the dark at 4 °C for 1 h. The secondary antibodies used were Alexa Fluor® 488 (Invitrogen, A21202; 1:1000), Alexa Fluor® 488 (Invitrogen, A21206; 1:1000), Alexa Fluor® 647 (Invitrogen, A21447; 1:2000) and Alexa Fluor® 647 (Jackson ImmunoResearch, 706-605-148; 1:2000). The cells were then washed in FACS buffer twice, washed again in DPBS twice and resuspended in DPBS and analyzed with the BDTM LSR II Flow Cytometer. Data analysis was performed using the FlowJo 7.0 software.

### RNA extraction, reverse transcription and quantitative PCR

PrepEase RNA Spin Kit (Affymetrix) was used to extract total RNA from differentiated hiPSCs according to the manufacturer’s instructions. To remove genomic DNA from the preparation, DNase treatment was carried out for 15 min at room temperature. Purified RNA was reverse transcribed using the High-Capacity cDNA Reverse Transcription Kit (Applied Biosystems). QPCR was performed on the CFX384 Touch™ Real-Time PCR Detection System with iTaq™ Universal SYBR*®* Green Supermix (Bio-Rad). Reported fold changes were based on relative expression values calculated using the 2-ΔΔC(T) method with normalization to actin expression for each sample. QPCR primers were custom-designed to span exon exon junction, wherever possible, using Primer-BLAST (NCBI). Sequences of primers used are listed in Supplementary Table 2. qPCR primers had been used for the following genes: *ABHD15*, *ACTB*, *ANKS4B*, *FGFR4*, *GLIS3*, *GLUT1*, *GLUT2*, *GLUT3*, *GPR39*, *HNF1A*, *HNF4A*, *INS*, *LAMA1*, *NRARP*, *PAK4*, *PDGFA*, *TM4SF4*, *TSPAN8*, *UGT2B4*.

### Western blotting

Cells were harvested by mechanical scraping on ice and lysed in M-PER (Thermo Scientific) in the presence of protease and phosphatase inhibitors (Sigma–Aldrich). Protein lysates were quantified using the BCA Assay (Thermo Scientific), separated with sodium dodecyl sulphate polyacrylamide gel electrophoresis (SDS-PAGE) using the Mini-PROTEAN Tetra Cell system (Bio-Rad) and transferred to PVDF membranes (Bio-Rad). Primary antibodies against endogenous HNF1A protein (Santa Cruz, sc-6548; 1:500), or actin (Sigma–Aldrich, A5441; 1:10000) were used, followed by HRP-conjugated secondary antibodies anti-goat IgG-HRP (Santa Cruz, sc-2354, 1:5000) and anti-mouse IgG-HRP (Bethyl labs, A90-516P, 1:10000). Chemiluminescent signals were detected using Super Signal West Dura Extended Duration substrate (Thermo Scientific).

### Stimulated insulin secretion assay

Glucose-stimulated insulin secretion (GSIS) was performed on hPSC-derived β-like cells. Briefly, approximately 100 β-like cell clumps were picked into each well of a 12-well non-treated cell culture plate (Eppendorf) and rinsed three times with the Krebs-Ringer Bicarbonate (KRB) buffer (129 mM NaCl, 4.8 mM KCl, 2.5 mM CaCl_2_, 1.2 mM MgSO_4_, 1.2 mM KH_2_PO_4_, 5 mM NaHCO_3_, 10 mM HEPES, 0.1% BSA in ddH2O, pH7.4 and sterile filtered). Samples were equilibrated in KRB buffer containing 2.8 mM D-glucose (Sigma–Aldrich) at 37 °C for 1 h. Samples were then incubated in KRB buffer containing 2.8 mM D-Glucose at 37 °C for 30 min and supernatants were collected. Next, cells were rinsed three times with PBS, incubated in KRB buffer containing 16.7 mM D-Glucose at 37 °C for 30 min and supernatants were collected again. At the end of the experiment, Mercodia Human Insulin ELISA kit (Mercodia) was used to measure the insulin content in the collected samples following manufacturer’s instructions. Human C-peptide ELISA kit (Mercodia) was used to measure the C-peptide content in the collected samples following manufacturer’s instructions.

Secretagogue-stimulated insulin secretion was performed on hPSC-derived β-like cells. ∼100 β-like cell clumps were picked into each well of a 12-well non-treated cell culture plate (Eppendorf) and rinsed three times with the Krebs-Ringer Bicarbonate (KRB) Buffer. Samples were equilibrated in KRB buffer containing 2.8 mM D-glucose (Sigma–Aldrich) at 37 °C for 1 h. Samples were then incubated in KRB buffer containing 2.8 mM D-Glucose at 37 °C for 30 min and supernatants were collected. Next, cells were rinsed three times with PBS, incubated in KRB buffer containing 10 uM ionomycin (Sigma–Aldrich)/100 µM glibenclamide (Sigma–Aldrich)/ 30 mM KCl (Merck) at 37 °C for 30 min and supernatants were collected again. At the end of the experiment, Mercodia Human Insulin ELISA kit (Mercodia) was used to measure the insulin content in the collected samples following manufacturer’s instructions.

### Mouse kidney capsule transplantation

All animal experiments have been done in compliance with the relevant ethical regulations and ethical approval from A*STAR with approval no. IACUC 181366. All relevant ethical regulations have been complied. Mice were housed under 12 h light/dark cycle at 25 °C and 60–80% humidity. The mice were placed on right lateral recumbence once they are anaesthetized for unilateral subrenal capsule injection. The shaved area was cleaned with 70% ethanol. The incision was started right below the most ventrolateral point of the vertebral column and extended ventrally to expose the lateral aspect of the left kidney. The kidney was gently lifted with sterile blunt-tipped surgical forceps to partially exteriorize it. A 24-gauge IV catheter was inserted at a shallow angle with respect to the renal surface at the injection site, into the renal parenchyma starting at its caudal pole advancing it until its tip is just below the renal capsule. 100–150 µl of cell suspension was injected. A visible bleb formed between the renal capsule and parenchyma. The injection site(s) were observed for potential leakage of the cell suspension and/or bleeding. The incision was then closed. After the procedure, the kidney was replaced into the abdominal cavity. The peritoneum and muscle tissue were opposed using 5-0 vicryl in a simple continuous pattern. The skin incision was closed with sterilized 7 mm stainless steel wound clips or using 5-0 vicryl in a simple interrupted pattern. After surgery, animals were observed until they can maintain sternal recumbency. Before being returned to the housing area, 0.1 mg/kg buprenorphine, SQ, was given on the opposite side from the incision or between the shoulder blades. Wound clips or remaining skin sutures were removed 7–14 days after placement.

### Glucose uptake assay

Approximately 100 hPSC-derived β-like cells were picked into each well of a 12-well cell culture plate and rinsed three times with PBS. They were then starved in 1 ml DMEM medium with no glucose (Gibco^TM^) for 4 h. The medium was removed and the β-like cells were rinsed three times with PBS. The cells were then exposed to DMSO or 30, 60, 90 or 120 μM fisetin and incubated in 1 ml DMEM medium containing 1 g/L D-glucose for 4 h before supernatants were collected. The cells were lysed using M-PER protein extraction reagent and measured for protein content. The supernatants were then measured for residual glucose amount using Glucose Uptake Colorimetric Assay Kit (Sigma–Aldrich). The level of glucose uptake was normalized to the protein content in each sample.

### Cellular ADP/ATP ratio assay

Approximately 100 hPSC-derived β-like cells were picked and rinsed three times with PBS. Cells were then lyzed and cellular ADP/ATP ratio was measured using ADP/ATP Bioluminescence Assay Kit (ApoSENSOR) (Biovision Incorporated), following manufacturer’s protocol.

In each well of a Corning^TM^ Solid White 96-well plate (ThermoFisher Scientific), 90 µl of Nucleotide Releasing Buffer and 10 µl of ATP monitoring enzyme were added. The reaction mix were left in the dark at RT for 1 to 2 h. The basal luminescence reading (Data A) was recorded at 630 nm with the GloMAX^®^96 Luminometer (Promega).

Approximately 100 hPSC-derived β-like cells were picked and rinsed three times with PBS. Cells were then treated with 50 µl of Nucleotide Releasing Buffer for 5 min before transferring onto a shaker at 50 rpm for 5 min. 50 µl of the mixture for each sample were transferred into one well of the 96-well plate, mixed thoroughly and left in the dark at room temperature for 2 min. Cellular ATP levels were recorded using the GloMAX machine (Data B). The readings were recorded again (Data C).

To each well of the 96-well plate, 1 µl of the ADP Converting Enzyme was added. The mixtures were mixed thoroughly and left at RT for 2 min. Cellular ADP level was then measured (Data D). The cellular ADP/ATP ratio was calculated using the formula below:

$${\rm{ADP}}/{\rm{ATP}}\,{\rm{ratio}}=\frac{{\mathrm{Data}}\,D-{\mathrm{Data}}\,C}{{\mathrm{Data}}\,B-{\mathrm{Data}}\,A}$$

### Generation of expression constructs

The pCDH plasmid (System Biosciences) containing the CMV promoter, N-terminal FLAG tag coding sequence and ampicillin resistance gene was used as the expression vector for *HNF1A*. *HNF1A* coding sequence was cloned from pLENTI-HNF1A (Origene). PCR products were inserted into the pCDH vector using the Quick Ligation Kit (NEB). The ligated plasmid was used to transform STBL3 competent cells (Thermo Fisher Scientific). Inserted sequences were verified by DNA sequencing. For site-directed mutagenesis, the p.H126D c.376 C > G mutation was generated using the following primers to replace a cytosine base to guanine base in the HNF1A coding sequence through a PCR using the Phusion polymerase (Thermo Scientific): Forward primer 5′ TACCTGCAGCAGGACAACATC 3′; Reverse primer: 5′ GATGTTGTCCTGCTGCAGGTA 3′, while the P291fsinsC mutation was generated using the following primers to introduce an additional cytosine base in the HNF1A coding sequence through a PCR using the Phusion polymerase (Thermo Scientific): Forward primer 5′ GGCCCCCCCCCAGGG 3′; Reverse primer: 5′ CCCTGGGGGGGGGCC 3′. The parental strand was digested following incubation with *Dpn1* (NEB). Introduced mutations were verified by DNA sequencing.

### Luciferase reporter assays

The h*GLUT2* promoter sequence (−520 to −1) was amplified from AD-293 cells using forward primer 3′ AGGTTATACTCCCCAGTAAAATG 5′ and reverse primer 3′ TGTACTAGTTGGGAGTCCTGTC 5′. The sequence was then cut with restriction enzyme EcoRV (NEB) to create blunt ends, and ligated into pGL4.10 vector. AD-293 cells were then co-transfected with the h*GLUT2* promoter construct, pRL-TK renilla vector and an overexpression vector (pCDH-GFP/ pCDH-HNF1A wild-type or mutants) using Lipofectamine 2000 (Life Technologies). Cells were transfected in triplicate wells and each experiment was independently performed three times. Cells were harvested 48 h after transfection, and luciferase activity was measured using the Dual Luciferase Assay System (Promega). Firefly luciferase activity was normalized to Renilla luciferase activity for each well.

### siRNA-mediated RNA interference

siRNA-mediated RNA interference was carried out on EndoC-βH1 cells using 100 nM non-targeting (D-001810-10) and *HNF1A*-targeting (J-008215-05-0002) ON-TARGETplus human siRNA (Dharmacon, GE Healthcare) with Lipofectamine RNAiMAX (Life Technologies) for 48 h before transfection for overexpression experiments. Cells were then transfected with an overexpression vector (pCDH-GFP/ pCDH-GLUT) using Fugene (Promega) for 48 h being harvested for GSIS and RNA extraction.

### RNA sequencing and differential expression analysis

Poly-A mRNA was enriched from 1 μg of total RNA with oligo-dT beads (Invitrogen). Up to 100 ng of poly-A mRNA recovered was used to construct multiplexed strand specific RNA-seq libraries as per manufacturer’s instruction (NEXTflex™ Rapid Directional RNA-SEQ Kit, dUTP-Based, v2). Individual library quality was assessed with an Agilent 2100 Bioanalyzer and quantified with a QuBit 2.0 fluorometer before pooling for sequencing on a HiSeq 2000 (1 × 101 bp read). The pooled libraries were quantified using the KAPA quantification kit (KAPA Biosystems) prior to cluster formation. Adapter sequences and low quality bases in Fastq read sequences were trimmed using Trimmomatic (v.0.33) (parameters: LEADING:3 TRAILING:3 SLIDINGWINDOW:4:15 MINLEN:36). The quality filtered Fastq sequence reads were then aligned to the human genome (hg19) using Tophat (v.2.0.14) (parameters:–no-coverage-search–library-type = fr-firststrand) and annotated with Ensembl gene IDs. The resulting bam files were used to generate feature read counts using the Python package-based htseq-count of HTSeq (v.0.6.1p1) (parameters: default union counting mode,–stranded = reverse). The read count matrix output from HTSeq was used to perform differential expression analysis using the edgeR package (available in R (v.3.1.3)) in both ‘classic’ and generalized linear model (glm) modes to contrast patient versus control. Procedures described in edgeR documentation were followed to calculate *P*-values, FDR adjusted *p*-values (q-values) and fold changes. A false discovery rate (FDR) cutoff of 0.05 and relative fold change >1.5 fold was used to filter significantly differentially expressed genes. These genes with Ensembl IDs were mapped to gene symbols.

### Chromatin immunoprecipitation (ChIP)

Approximately 200–300 hPSC-derived endocrine progenitors clumps were dissociated using TrypLE^TM^ Express Enzyme cross-linked with 3.3 mg/ml of dimethyl 3,3’-dithiobispropionimidate and 1 mg/ml of 3,3’-dithiodipropionic acid di(Nhydroxysuccinimide ester) (both Sigma–Aldrich) for 30 min at room temperature and with 1% formaldehyde (Amresco) for 15 min. The cross-linking reaction was quenched with 0.125 M glycine and cells were first lysed in cell lysis buffer (10 mM Tris-HCl pH 8, 10 mM NaCl and 0.2% NP-40) and then in nuclear lysis buffer (50 mM Tris-HCl pH 8, 10 mM EDTA and 1% SDS) on ice in the presence of protease inhibitors on ice. Nuclear lysates were diluted in IP dilution buffer (20 mM Tris-HCl pH 8, 2 mM EDTA, 150 mM NaCl, 0.01% SDS and 1% Triton X-100) and sonicated for 30 s on/45 s off for 12 cycles using a Q500 sonicator (QSonica) with microtip probes at 30% power. Sonicated samples were pre-cleared using 10 μg rabbit IgG (Santa Cruz) and Protein A/G agarose beads. Agarose beads were removed by centrifugation and a portion of the supernatant was collected as the input control. Samples were divided equally and incubated with 10 μg of HNF1A antibody (Abcam ab96777) or rabbit IgG overnight at 4 °C. The following day, samples were incubated with Protein A/G agarose beads and the beads were recovered and washed with IP wash buffer and Tris-EDTA buffer. The immunoprecipitated DNA was eluted from the beads using IP elution buffer (100 mM NaHCO_3_, 1% SDS, 100 mM DTT). Samples were successively treated with RNaseA, NaCl and Proteinase K. DNA was extracted by phenol/chloroform extraction. Finally, qPCR was carried out on the input, HNF1A pulldown and IgG samples using SYBR green (Bio-Rad), targeting the *GLUT2* promoter or a control region in *GAPDH*. QPCR data were quantitated using a standard curve based on the input DNA, and normalized against *GAPDH*. Results are expressed as fold change for *HNF1A* pulldown relative to IgG control.

### ChIP sequencing and analysis

#### Library preparation

Library preparation was done using a commercially available kit, NEBNext® Ultra™ DNA Library Prep Kit (NEB #E7645) for Illumina® following the manufacturer’s protocol. During the library construction, the ChIP-ed DNA underwent End Repair, Adapter ligation, no size selection and PCR enrichment to generate the final sequencing ready library. The quality of the library was checked using Agilent D1000 ScreenTape. A single peak in the expected (depending on the ChIP fragmentation) should be observed indicating that the library was good and suitable for sequencing. The different libraries were then pooled together and QC using Agilent high sensitivity DNA kit and KAPA quantification.

#### Cluster generation and sequencing

The sample were linearized with 0.2 N NaOH into single-stranded forms, they were then neutralized and diluted into 4pM loading concentration with Hybridization buffer (HT1). The NEXTSEQ High Output was performed using the Illumina NEXTSEQ 500 Sequencers with the Illumina® Reagent v2 (75 cycle kit) Kit. The DNA was attached to the flowcell surfaces and amplified to clusters, followed by attachment with the Sequencing primers and run at 1 × 76cycles, generating Single-Read 75 base-pair reads. The images were captured by the NextSeq Control Software (NCS), and the Real Time Analysis (RTA) software converted the images into Basecall (bcl) files. All the bcl files were then transferred to the server for storage and primary analysis.

#### Primary analysis

In the primary analysis, the bcl files were converted into fastq files using the bcl2fastq. After the conversion, the fastq reads were filtered to remove all the reads that did not pass filtering, leaving only useable Passed Filtered (PF) reads. The primary analysis result was then generated as the Demultiplexed_Stats file and reviewed, and the PF fastq files were then passed on for further analysis. The quality of the reads is determined by Q-score calculation of the bcl2fastq software and attached PDF have the details. The data are only passed if the Q30 is above 75%, in this case all three libraries have passed.

#### ChIP-seq analysis

Raw Fastq reads were processed with Trimmomatic (v0.36) in single end mode with settings TruSeq3-SE.fa:2:30:10 LEADING:3 TRAILING:3 SLIDINGWINDOW:4:15 MINLEN:36 to remove sequencing adapters. Trimmed reads were mapped to the human reference genome (hg19) using the bowtie2 program^95^ with default settings. Aligned reads were then analyzed using the Homer suites of programs^96^. Peaks were identified using the findPeaks program with default settings (-style factor). Peaks were annotated with the annotatePeaks.pl script to assign the peaks to the nearest transcription start site (TSS) of each gene (Poisson *p*-value < 1e-04, Fold over input required: 4). Motif analysis performed with findMotifsGenome.pl (-size 200 -mask).

### Statistical analysis

Statistical analyses were performed using GraphPad Prism 6 and Microsoft Excel 2016. The results are expressed as the mean ± standard error of mean (SEM). Simple linear regression analysis was used to determine the direction of the data trend. The regression trend is expressed using Pearson’s correlation coefficient (R^2^). Unpaired Students *t*-test for groups assuming equal variance was performed (unless specified otherwise) to determine *p*-values. In figures asterisk indicates *P*-value < 0.05, n.s. *p* > 0.05.

### Reporting summary

Further information on research design is available in the Nature Research Reporting Summary linked to this article.

## Supplementary information

Supplementary Information

Reporting Summary

Description of Additional Supplementary Files

Supplementary Movie 1

Supplementary Movie 2

Supplementary Data 1

Supplementary Data 2

Supplementary Data 3

Supplementary Data 4

Supplementary Data 5

## Data Availability

Data reported in this paper were deposited into Gene Expression Omnibus (GEO) accessible via accession numbers GSE140208 (RNA-Seq) and GSE139832 (ChIP-Seq). Crystal structure of WT HNF1A–DNA complex is available in Protein Data Bank (PDB 1IC8). Source data are provided with this paper.

## Source data

Source Data
