# Supplementary material for: Decreased GLUT2 and glucose uptake contribute to insulin secretion defects in MODY3/HNF1A hiPSC-derived mutant β cells
Source: Nat Commun. 2021 May 25;12:3133. doi: 10.1038/s41467-021-22843-4 (PMC8149827; doi:10.1038/s41467-021-22843-4)
Supplement: Supplementary file 1 — Supplementary Information [file 41467_2021_22843_MOESM1_ESM.pdf]

SUPPLEMENTARY INFORMATION

Supplementary Table 1. Key Resources.

REAGENT or RESOURCE	SOURCE	IDENTIFIER
**Antibodies**		
Goat anti-OCT4	Santa Cruz	sc-8628 RRID:AB_653551
Rabbit anti-SOX2	Abcam	ab97959 RRID:AB_2341193
Goat anti-NANOG	R&D Systems	AF1997 RRID:AB_355097
Mouse anti-SSEA4	STEMCELL Technologies	60062AD RRID:AB_2721031
Mouse anti-TRA-1-60	STEMCELL Technologies	60064AD RRID:AB_2686905
Mouse anti-ACTB (β-actin)	Cell Signaling Technology	3700S RRID:AB_2242334
Rat anti-C-peptide	Developmental Studies Hybridoma Bank	GN-ID4 RRID:AB_2255626
Mouse anti-GATA4	Thermo Fisher Scientific	MA5-15532 RRID:AB_10989032
Rabbit anti-GLUT2	Abcam	ab95256 RRID:AB_10859605
Rabbit anti-GLUT2	Santa Cruz	sc-9117 RRID:AB_641068
Rabbit anti-HNF1A	Abcam	ab96777 RRID:AB_10679303
Goat anti-HNF1A	Santa Cruz	sc-6547 RRID:AB_648295
Goat anti-HNF1A	Santa Cruz	sc-6548 RRID:AB_648293
Rabbit anti-HNF1A	Abcam	ab204306
Guinea pig anti-INS	Abcam	ab7842 RRID:AB_306130
Goat anti-NKX6.1	LifeSpan BioSciences	LS-C124275 RRID:AB_10805839
Guinea pig anti-PDX1	Abcam	ab47308 RRID:AB_777178
Goat anti-PDX1	R&D Systems	AF2419 RRID:AB_355257
**Chemicals, Peptides, and Recombinant Proteins**		
Dulbecco's High Glucose Modified Eagles Medium	HyClone™	#SH30022FS
DMEM medium with no glucose	Gibco™	11966025
DMEM/F12 with GlutaMAX supplement, Pyruvate	Invitrogen	10565042
GE Healthcare Fetal Bovine Serum	HyClone™	11521851
KnockOut Serum Replacement (KOSR)	Invitrogen	10828028
BSA	Sigma-Aldrich	A9418

REAGENT or RESOURCE	SOURCE	IDENTIFIER
GlutaMAX™ Supplement	Gibco™	11574466
Non-essential amino acids (NEAA)	Invitrogen	11140050
Beta-mercaptoethanol (1000X)	Gibco™	21985023
Trypsin-EDTA 0.25%	Gibco™	25300054
ReLeSR™	STEMCELL Technologies	05873
TrypLE™ Express Enzyme	ThermoFisher	12605010
Fibronectin	Sigma-Aldrich	F0895
Fisetin	MERCK	F4043
ECM	Sigma-Aldrich	E1270
D-Glucose	Sigma-Aldrich	G8769
Gelatin	MERCK	ES-006-B
Glibenclamide	Sigma-Aldrich	G0639
Ionomycin calcium salt	Sigma-Aldrich	I0634
L-Ascorbic acid (vitamin C)	Sigma-Aldrich	A92902
Penicilin/Streptomycin	Gibco™	15140122
Potassium Chloride	Merck	7447-40-7
Y-27632	STEMCELL Technologies	72302
FGF2	MACS	130-093-840
Activin A	R&D Systems	338-AC-50
CHIR-99021	Tocris	4423
BMP4	STEMCELL Technologies	02524
SB431542	Abcam	AB120163
FGF7	MACS	130-097-178
SANT-1	Santa Cruz	sc-203253
LDN193189	Sigma	SML0559-5MG
RA	Sigma	D2650-10
LY294002	LC Labs	L-7962
LDN	Stemgent	04-0019
ITS-X	Thermo Fisher Scientific	51500-056
PDBu	Tocris	4153
T3	Millipore	642511

REAGENT or RESOURCE	SOURCE	IDENTIFIER
ALK5 inhibitor II	Enzo Life Sciences	ALX-270-445
Heparin	Sigma-Aldrich	H3149
Transferrin	Sigma-Aldrich	T8158-100MG
Sodium selenite	Sigma-Aldrich	214485-100G
hBTC	Cell Signaling Techonologies	5235SF
XXI	Millipore	565790
**Commercial Assays**		
Nucleospin® RNA kit	Macherey-Nagels	740955.250
iTaq™ Universal SYBR® Green Supermix	Bio-Rad Laboratories	1725125
High-Capacity cDNA Reverse Transcription Kit	Applied Biosystems	4368814
Glucose Uptake Colorimetric Assay Kit	Sigma-Aldrich	MAK083
ADP/ATP Bioluminescence Assay Kit (ApoSENSOR)	Biovision Incorporated	K255
Mercodia Insulin ELISA	Mercodia Immunoassays and Services	10-1113-01
C-peptide ELISA	Mercodia	10-1136-01
Dual Luciferase Assay System	Promega	E1980
**Deposited Data**		
RNA-sequencing	This paper	GSE140208
ChIP-sequencing	This paper	GSE139832
**Experimental Models: Cell Lines**		
H9 hESC line	WiCell Research Institute	NIHhESC-10-0062 RRID:CVCL_9773
AD-293 line	Agilent	240085 RRID:CVCL_KA63
EndoC-βH1 line	Univercell Biosolutions	EndoC-βH1 RRID:CVCL_L909
Fibroblast (to generate iAGb)	Coriell Institute	AG16102 RRID:CVCL_2G48
CF-1 MEF	Gibco	A34181 RRID:CVCL_5251
**Experimental Models: Organisms/Strains**		
NOD-SCID mice	Taconic Biosciences	NOD/MrkBomTac-*Prkdc*^{scid} RRID:IMSR_TAC:nodsc
**Software and Algorithms**		

REAGENT or RESOURCE	SOURCE	IDENTIFIER
PRISM v8 graphing and statistical software	GraphPad	http://www.graphpad.com/scientific-software/prism/ RRID:SCR_002798
FlowJo 7.0 software	BD Biosciences	https://www.flowjo.com/solutions/flowjo/ RRID:SCR_008520

Supplementary Table 2. Quantitative real-time PCR primers used in this study.

Gene	Accession Number	Forward Primer (5' to 3')	Reverse Primer (5' to 3')
ABHD15	NM_198147	GCTGTTCGCGGTGAGCGAAG	GGCATACCTGCTGAGGGCGAT
ACTB	NM_001101	TTGCCGATCCGCCGCCCGTC	CCCATGCCCACCATCACGCCCTG
ANKS4B	NM_145865	TGGCCAAGTTCTGGTGAGCAGG	GGGTCCCCTCCTCTACTGCAGATT
FGFR4	NM_002011	GGAGCGCTCGGGCTGTCTG	TGCCTTCCTGGCTCCTCCTCA
GLIS3	NM_001042413	CGGACGCATCTGGACACCAAAC	GGGACTGCACGGTGAGGCAA
GLUT1	NM_006516	AGCAGCAAGAAGCTGACGGGTCGCC	AGCGTGGTGAGCGTGGTGGGC
GLUT2	NM_000340	TTGGTGGGTGGCTTGGGGAC	ACCAGGCCTGAAATTAGCCCACA
GLUT3	NM_006931	GCTAAGCAGATCCTCCAGCGGT	ATTAACCACACCCGCGCCGA
GPR39	NM_001508	GCAGACCATCATCTTCCTGAGGCT	GTCGTGCTTGGGTTTGGCCG
HNF1A	NM_001306179	CTTCTGCAGGAGGACCCGTGGCGT	GGCGGCCCGCTTCTGCGTCT
HNF4A	NM_178849	GGACGACCAGGTGGCCCTGCTCAGA	GCTCCGGGCAGTGCCGAGGGA
INS	NM_000207	CCTGCAGGTGGGGCAGGTGGAGC	CGGGTGTGGGGCTGCCTGCG
LAMA1	NM_005559	ACAGCGCAAACCCCAGAGAACGCCA	CCAGGTCGAGGGGCATTGGCAGC
NRARP	NM_001004354	TGGGTGGAGTTTGTGCGCCT	CACCATCAGGCTGGGCGGTA
PAK4	NM_005884	AGTTAGGCCGCGAGCGACTG	GTGCGGCCTGGTCTGATGCT
PDGFA	NM_002607	CGGGCCGCGCTCCCTAAG	CGGCTTCCTCGGCCAGAACA
TM4SF4	NM_004617	CGATTTGCGATGTTCACCTCCACG	GAGGCTCTCGGCACTTGTTCCA
TSPAN8	NM_004616	AGTCGCTGCATGCTTCTGTTGTTTT	CCCCTGTGGCGCTCAAAAGC
UGT2B4	NM_021139	TGCCAAACCCCTACCGAAGGAAA	TGTGGGATCTTGGCAAGGGCT

Figure S1: Low et al.,

a

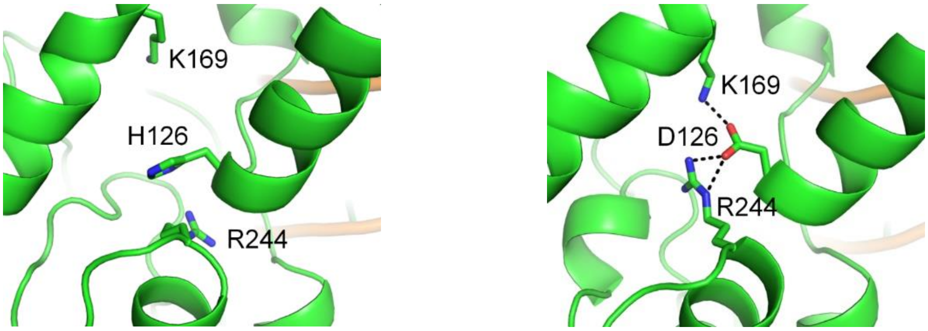

Supplementary Figure 1. Interactions of residue 126 in HNF1A–DNA complex. Crystal structure of WT HNF1A–DNA complex (PDB 1IC8) (Chi et al., 2002). Final trajectory structure obtained from accelerated molecular dynamics (aMD) simulation of mutant HNF1A H126D–DNA complex.

Figure S2: Low et al.,

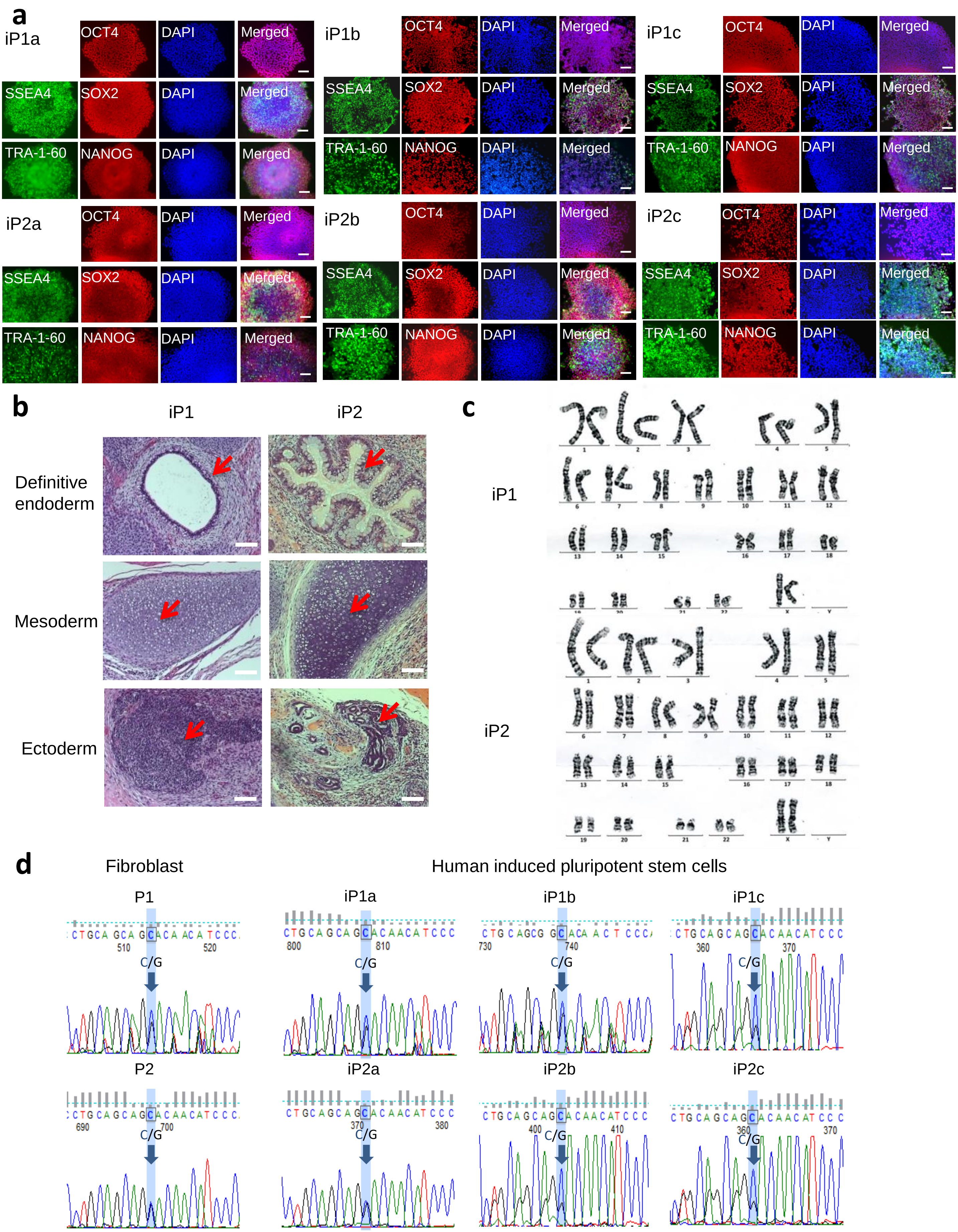

Supplementary Figure 2. Characterization of MODY3-hiPSCs. (a) Immunohistochemistry stain for pluripotency markers OCT4 (red), SOX2 (red), SSEA4 (green), NANOG (red) and TRA-1-60 (green) and nuclear stain using DAPI (blue). n = 3 independent experiments. (scale bar:100μM). (b) Hematoxylin and eosin (H&E) stain of teratoma tissue section formed by patient-derived hiPSCs. Presence of three germ layers (indicated by red arrows): definitive endoderm, mesoderm, ectoderm. n = 1 independent experiment. (scale bar: 100 μm). (c) Karyotyping analysis confirmed normal karyotype of hiPSCs derived from P1 and P2. (d) Cycle sequencing confirmed the presence of heterozygous *HNF1A*$^{+/H126D}$ mutation (highlighted) in patient-derived hiPSCs. Cytosine in blue, thymine in red, guanine in black, adenine in green. hiPSCs: human induced pluripotent stem cells. iP1a-c: hiPSC lines generated from P1, iP2a-c: hiPSC lines generated from P2. P1: patient 1, P2: patient 2.

Figure S3: Low et al.,

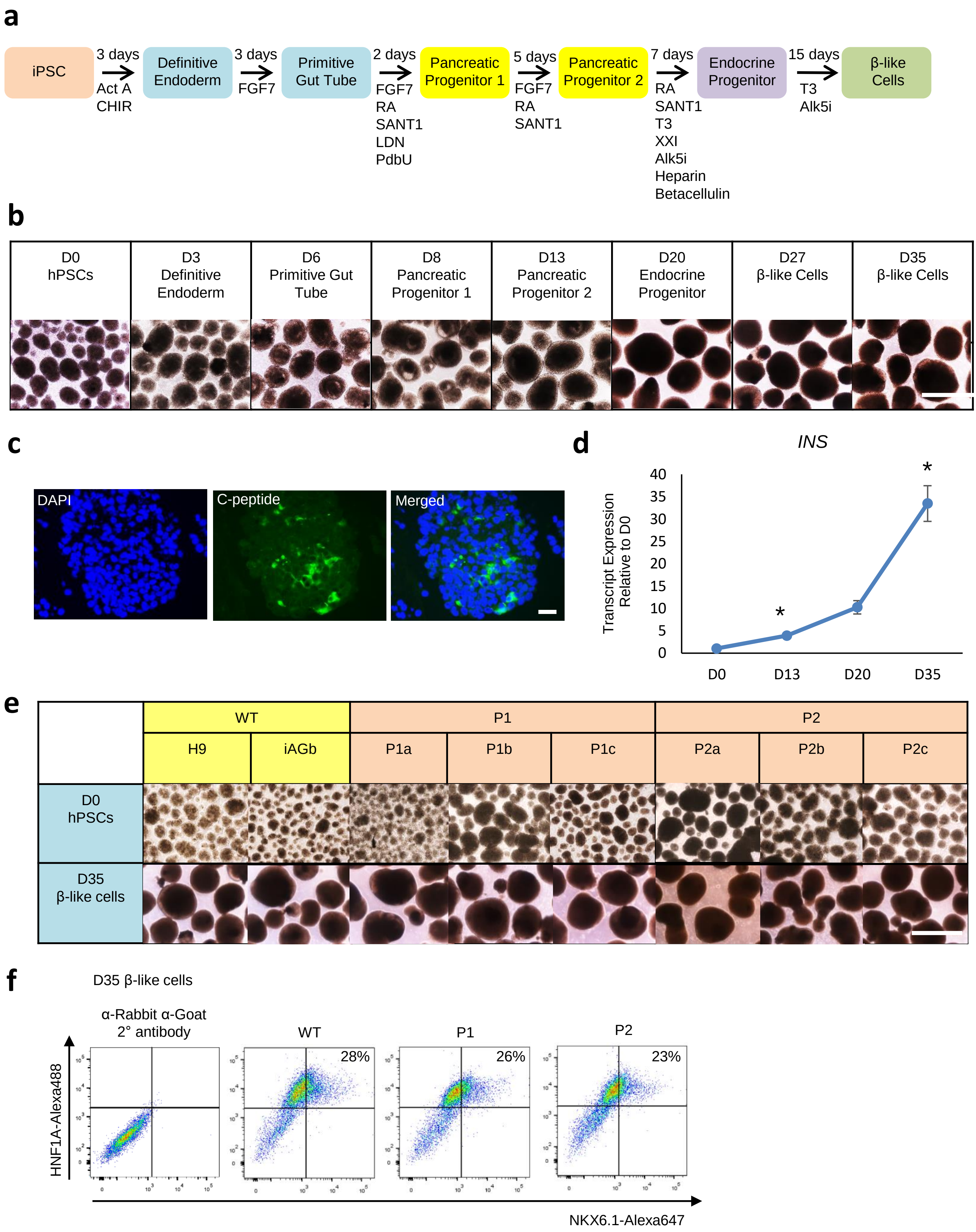

Supplementary Figure 3. Differentiation of MODY3 patient-specific hiPSCs along the pancreatic lineage. (a) Schematic of differentiation protocol and growth factors added at each stage of pancreatic differentiation. (b) Cell clusters at various stages during the 35-day pancreatic differentiation. n = 3 independent experiments. (scale bar: 100 µm). (c) Immunohistochemistry stain for C-peptide protein (green) and nuclear stain using DAPI (blue) in H9 cell clusters at day 35 of pancreatic differentiation. n = 3 independent experiments. (scale bar: 50 µm). (d) RT-qPCR analysis of *INS* transcripts in H9 during various stages of pancreatic differentiation. n = 3 independent experiment. Error bars represent standard error of mean (SEM). (e) WT and mutant cell clusters at the start and end of the 35-day pancreatic differentiation. n = 3 independent experiments. (scale bar: 100 µm). (f) Flow cytometry analysis of HNF1A and NKX6.1 proteins in D35 β-like cells. D: day of differentiation. hPSCs: human pluripotent stem cells. P1a-c: cell lines generated from P1, P2a-c: cell lines generated from P2. WT: wild type, P1: patient 1, P2: patient 2. For all statistical analysis: Error bars represent standard error of mean (SEM). Unpaired one-tailed Student's t-test was performed. * indicates P-value < 0.05 compared to D0. Source data are provided as a Source data file.

Figure S4: Low et al.,

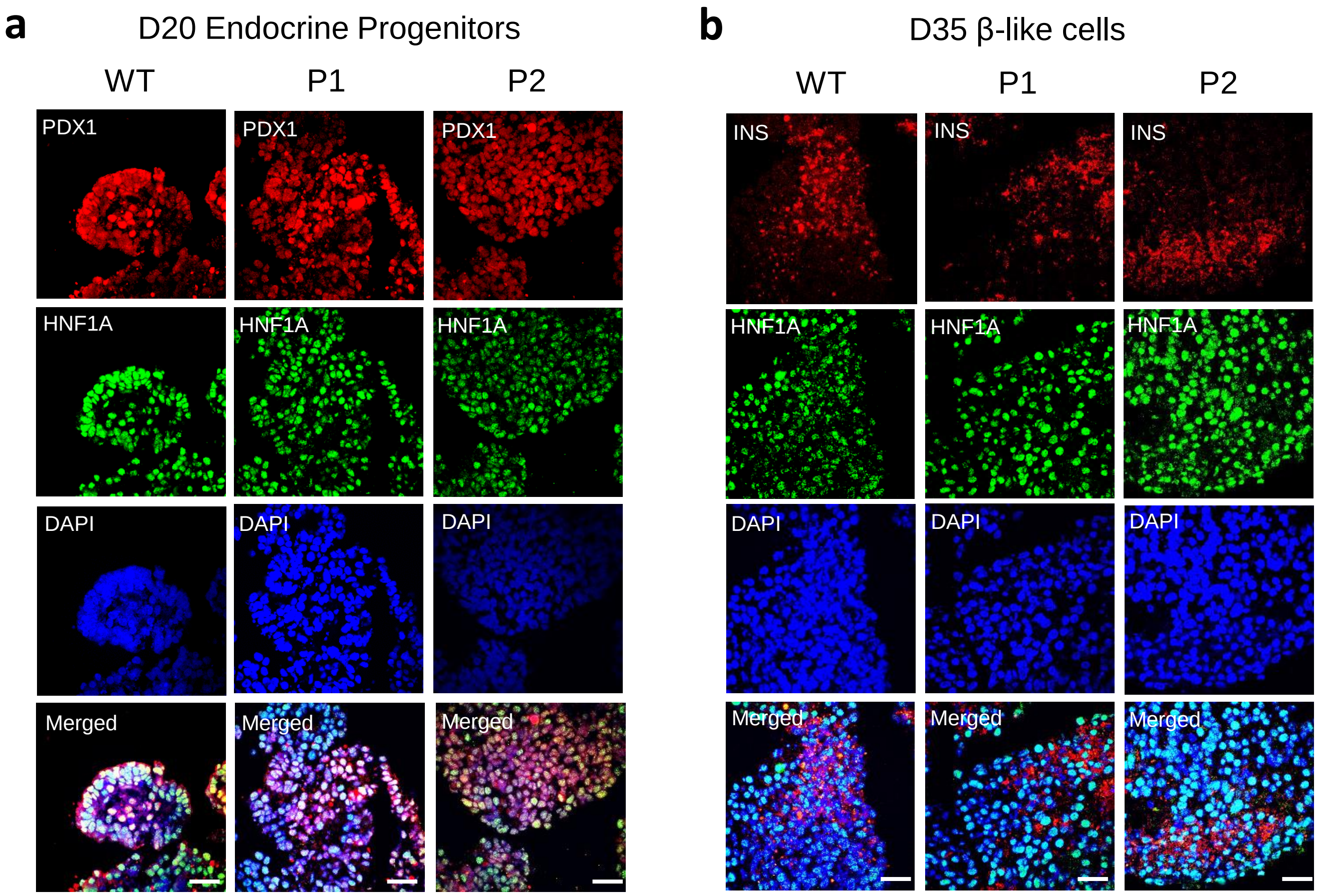

Supplementary Figure 4. Comparison of pancreatic differentiation between WT and MODY3-hiPSCs. (a) Immunohistochemistry stain for HNF1A protein (green), PDX1 protein (red) and nuclear stain using DAPI (blue) in WT, P1 and P2 endocrine progenitor cell clusters at day 20 of pancreatic differentiation. n = 3 independent experiments. (scale bar: 100 µm). (b) Immunohistochemistry stain for HNF1A protein (green), INS protein (red) and nuclear stain using DAPI (blue) in WT, P1 and P2 D35 β-like cell clusters at day 35 of pancreatic differentiation. n = 3 independent experiments. (scale bar: 100 µm). D: day of differentiation. WT: wild type, P1: patient 1, P2: patient 2. hiPSCs: human induced pluripotent stem cells. Source data are provided as a Source data file.

Figure S5: Low et al.,

a

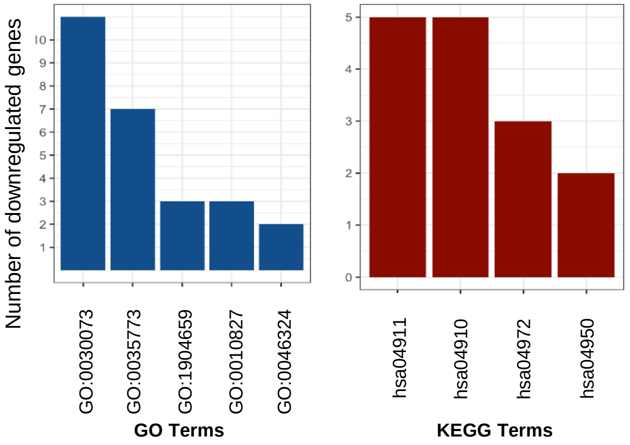

b

GO/KEGG ID	Description	Downregulated Genes	P-value
GO:0030073	insulin secretion	*PTPRN2, BAIAP3, LRP5, MAFA, PHPT1, LRP1, TRPM4, PFKL, RAB11B, ARL2, ITPR3*	0.002
GO:0035773	insulin secretion involved in cellular response to glucose stimulus	*PTPRN2, BAIAP3, LRP5, PHPT1, LRP1, TRPM4, RAB11B*	0.012
GO:1904659	glucose transmembrane transport	*RNASEL, TRIB3, ASPSCR1*	0.214
GO:0010827	regulation of glucose transmembrane transport	*RNASEL, TRIB3, ASPSCR1*	0.295
GO:0046324	regulation of glucose import	*RNASEL, ASPSCR1*	0.512
hsa04911	Insulin secretion Pathway	*RAB3A, ATP1A3, TRPM4, CREB3L1, ITPR3*	0.177
hsa04910	Insulin signalling pathway	*RPTOR, MAP2K2, PCK1, PCK2, FASN*	0.353
hsa04972	Pancreatic secretion	*SLC4A4, ATP1A3, ITPR3*	0.042
hsa04950	Maturity onset diabetes of the young	*MAFA, IAPP*	0.060

c

Genes associated with insulin secretion, glucose metabolism and diabetes

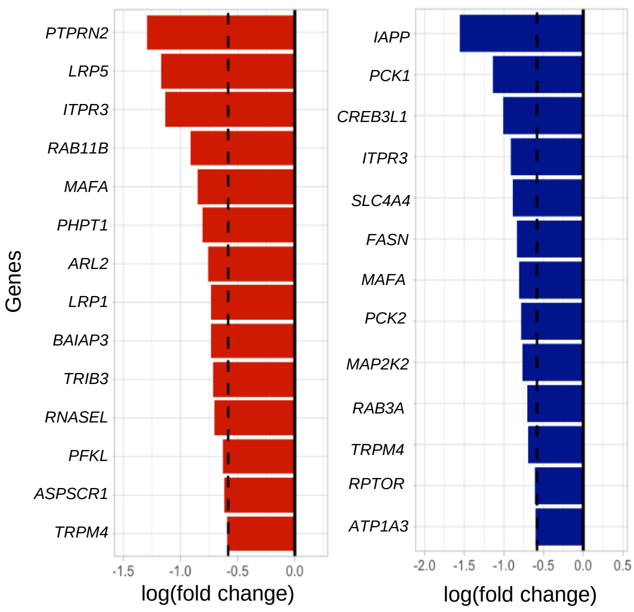

d

GO and KEGG analysis of overlapping downregulated gene in *HNF1A*$^{+/H126D}$ β-like cells and *HNF1A*$^{+/T260M}$ islets

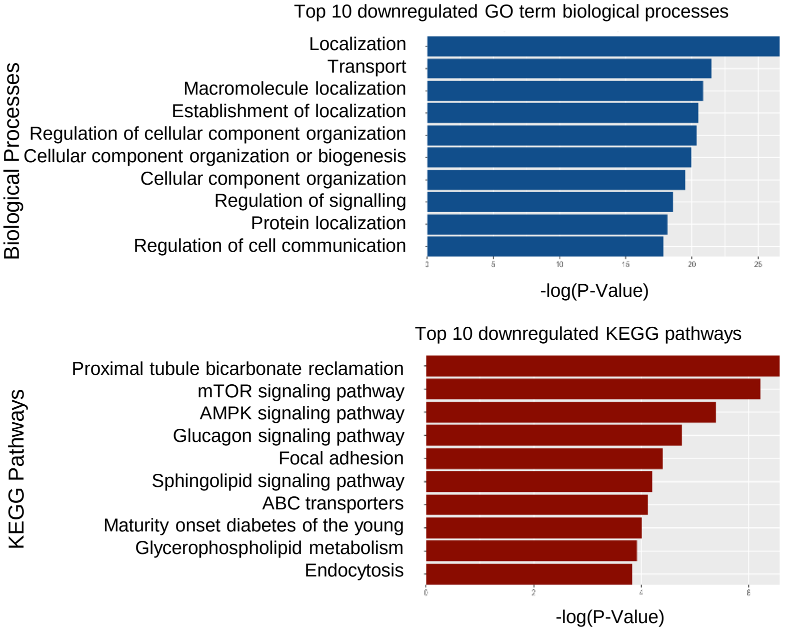

Supplementary Figure 5. Bioinformatics analyses of *HNF1A* transcriptomics datasets. (a) Number of gene candidates that are downregulated in *HNF1A*$^{+/H126D}$ endocrine progenitors that are associated with insulin secretion, glucose metabolism and diabetes according to Gene Ontology (GO) and Kyoto Encyclopedia of Genes and Genomes (KEGG) annotation. Unadjusted P-value were calculated using two-sided Mann-Whitney U test according to the GOSeq protocol (Young et al., 2010). (b) Table showing the details of each GO and KEGG annotation along with the list of gene candidates and P value. Unadjusted P-value were calculated using two-sided Mann-Whitney U test according to the GOSeq protocol (Young et al., 2010). (c) Transcript fold change of gene candidates that are downregulated in *HNF1A*$^{+/H126D}$ endocrine progenitors that are associated with insulin secretion, glucose metabolism and diabetes based on RNA-Seq analysis. Black dotted line indicates cut-off of 1.5-fold. (d) GO and KEGG analysis of the 347 genes commonly downregulated in both *HNF1A*$^{+/H126D}$ endocrine progenitors and *HNF1A*$^{+/T260M}$ islets. Bar chart showing the top 10 GO biological processes and KEGG pathway affected, ranked by ascending order of p value. Unadjusted P-value were calculated using two-sided Mann-Whitney U test according to the GOSeq protocol (Young et al., 2010). ChIP-Seq: Chromatin immunoprecipitation sequencing. WT: wild type.

Figure S6: Low et al.,

a CHIP-Seq motif ranking for WT endocrine progenitors

* - possible false positive

Rank	Motif	P-value	log P-pvalue	% of Targets	% of Background
1	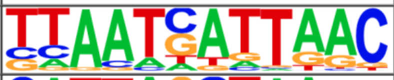	1e-229	-5.281e+02	57.38%	1.53%
2	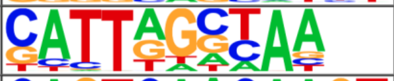	1e-16	-3.773e+01	22.62%	7.34%
3	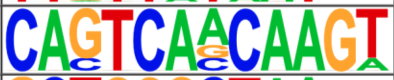	1e-13	-3.155e+01	2.30%	0.01%
4	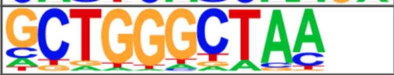	1e-12	-2.884e+01	7.87%	1.14%
5	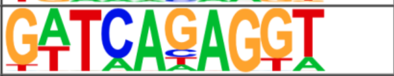	1e-12	-2.841e+01	5.57%	0.49%
6 *	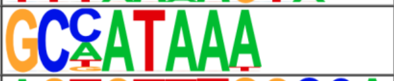	1e-11	-2.665e+01	10.82%	2.49%
7 *	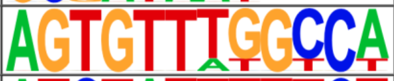	1e-10	-2.324e+01	1.64%	0.01%
8 *	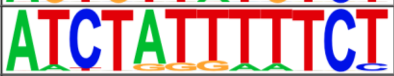	1e-9	-2.267e+01	3.28%	0.16%
9 *	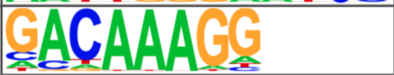	1e-9	-2.150e+01	24.59%	11.79%
10 *	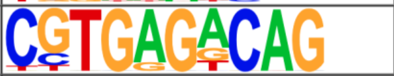	1e-8	-2.015e+01	3.28%	0.21%
11 *	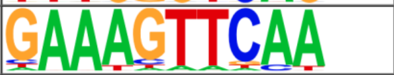	1e-8	-1.888e+01	5.90%	1.05%
12 *	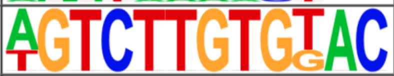	1e-7	-1.701e+01	0.98%	0.00%
13 *	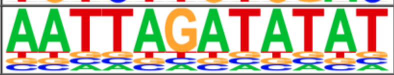	1e-1	-3.693e+00	0.33%	0.01%

b CHIP-Seq motif ranking for *HNF1A*$^{+/H126D}$ endocrine progenitors

* - possible false positive

Rank	Motif	P-value	log P-pvalue	% of Targets	% of Background
1	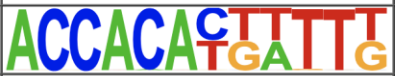	1e-12	-2.782e+01	11.54%	0.06%
2 *	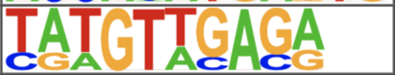	1e-10	-2.366e+01	23.08%	1.67%
3 *	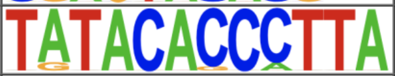	1e-8	-2.033e+01	5.77%	0.00%
4 *	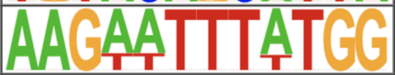	1e-8	-1.989e+01	7.69%	0.03%
5 *	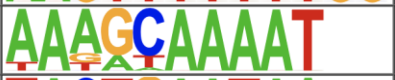	1e-7	-1.736e+01	21.15%	2.34%
6 *	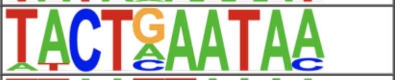	1e-7	-1.691e+01	15.38%	0.99%
7 *	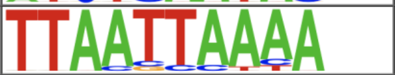	1e-7	-1.652e+01	19.23%	1.98%
8 *	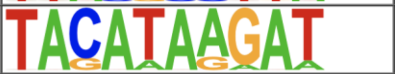	1e-6	-1.598e+01	11.54%	0.43%
9 *	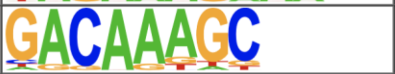	1e-6	-1.444e+01	28.85%	6.37%
10 *	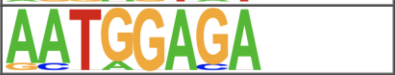	1e-4	-1.042e+01	19.23%	3.92%

Supplementary Figure 6. ChIP-Seq analyses of motifs enriched in the HNF1A-bound regions in WT and MODY3 endocrine progenitors. Motifs enriched in the HNF1A-bound regions of (a) WT endocrine progenitors and (b) *HNF1A*$^{+/H126D}$ endocrine progenitors. Cumulative Poisson p-value was determined according to protocol specified in the HOMER software suite (v4.11) using findMotifsGenome.pl. (Heinz et al., 2010).

Figure S7: Low et al.,

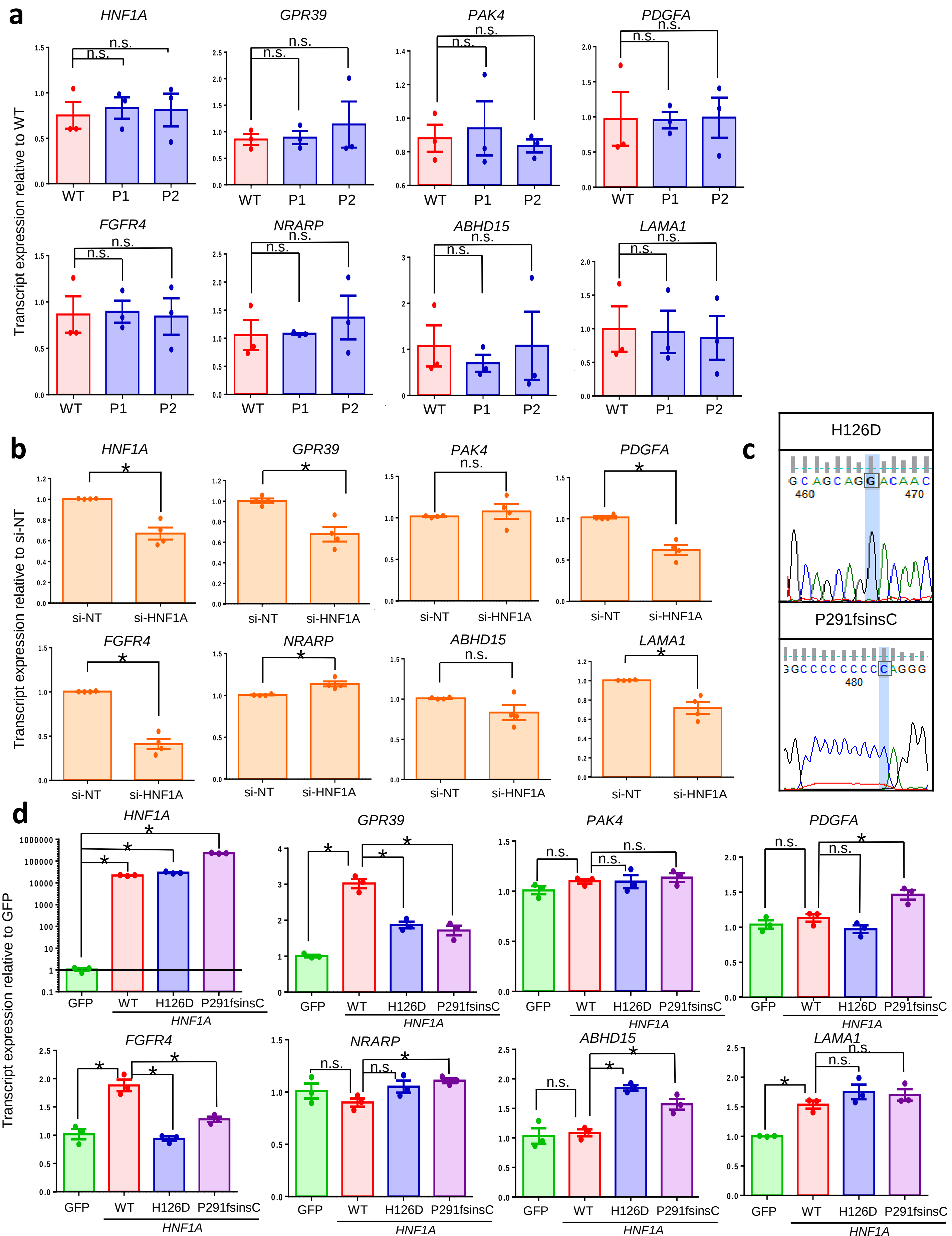

Supplementary Figure 7. Evaluation of putative HNF1A targets affected by *HNF1A*$^{+/H126D}$ mutation based on RNA-Seq and ChIP-Seq analyses. RT-qPCR analysis of candidate genes in (a) WT (red) and mutant (blue) hPSC-derived endocrine progenitors. n = 3 independent experiments. or (b) EndoC-βH1 cells transfected with *HNF1A* siRNA (si-HNF1A) and non-targeting siRNA as negative control (si-NT). n = 3 independent experiments. P-value for *HNF1A* = 0.0102, *GPR39* = 0.0151, *PDGFA* = 0.0051, *FGFR4* = 0.0018, *NRARP* = 0.0220, *LAMA1* = 0.0180. (c) Cycle sequencing confirmed successful site directed mutagenesis nucleotide change (highlighted) to generate *HNF1A* constructs containing p.H126D c.376C>G and P291fisinsC mutations. Cytosine in blue, thymine in red, guanine in black, adenine in green. (d) RT-qPCR analysis of candidate genes in AD-293 cells overexpressed with GFP (green) and various WT (red), H126D (blue) and P291fsinsC (purple) *HNF1A* constructs. n = 3 independent experiments. P-value for *HNF1A* = 0.0007 (WT), 0.0034 (H126D), 0.0006 (P291fsinsC); *GPR39* = 0.0025 (WT), 0.0028 (H126D), 0.0023 (P291fsinsC); *PDGFA* = 0.0229 (P291fsinsC); *FGFR4* = 0.0034 (WT), 0.0056 (H126D), 0.0155 (P291fsinsC); *NRARP* = 0.0178 (P291fsinsC); *ABHD15* = 0.0007 (H126D), 0.01580 (P291fsinsC); *LAMA1* = 0.0141 (WT). GFP: green fluorescent protein. WT: wild type, P1: patient 1, P2: patient 2. RT-qPCR: quantitative reverse transcription polymerase chain reaction. hPSCs: human pluripotent stem cells. WT: wild type, P1: patient 1, P2: patient 2. For all statistical analysis: Error bars represent standard error of mean (SEM). Unpaired one-tailed Student's t-test was performed. * indicates P-value < 0.05. Source data are provided as a Source data file.

Figure S8: Low et al.,

a

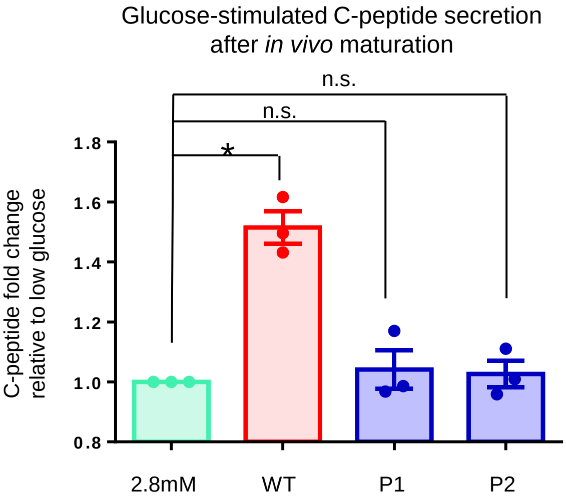

b

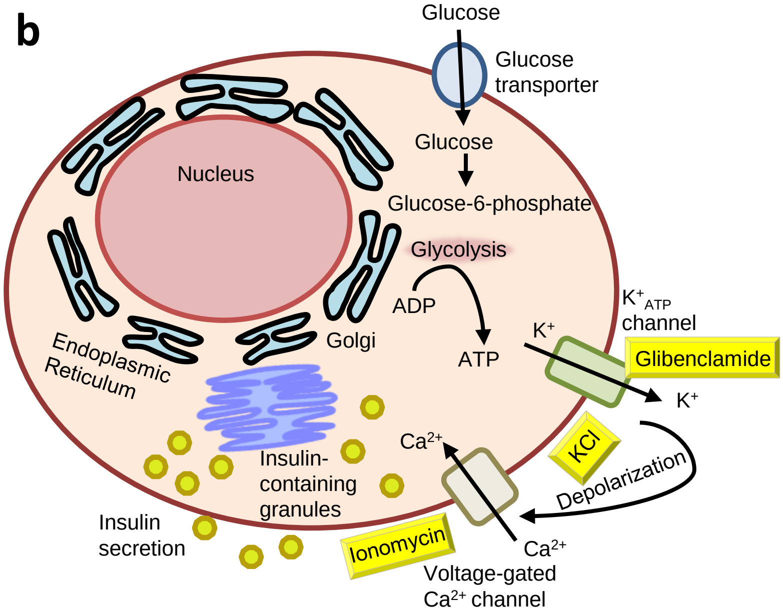

Supplementary Figure 8. Stimulus-secretion coupling in β cell. (a) Glucose-stimulated C-peptide secretion of WT (red) and patient-specific (blue) hPSC-derived β-like cells after *in vivo* maturation in mouse kidney capsule for 23 weeks. All C-peptide fold changes are normalized to C-peptide amounts secreted at 2.8 mM glucose (green) under each condition. n = 3 independent experiments; $p = 0.0006$ (WT). (b) Diagram of stimulus-secretion coupling in human β cell. WT: wild type, P1: patient 1, P2: patient 2. hPSCs: human pluripotent stem cells. For all statistical analysis: Error bars represent standard error of mean (SEM). Unpaired one-tailed Student's t-test was performed. * indicates P-value < 0.05. Source data are provided as a Source data file.

Figure S9: Low et al.,

a

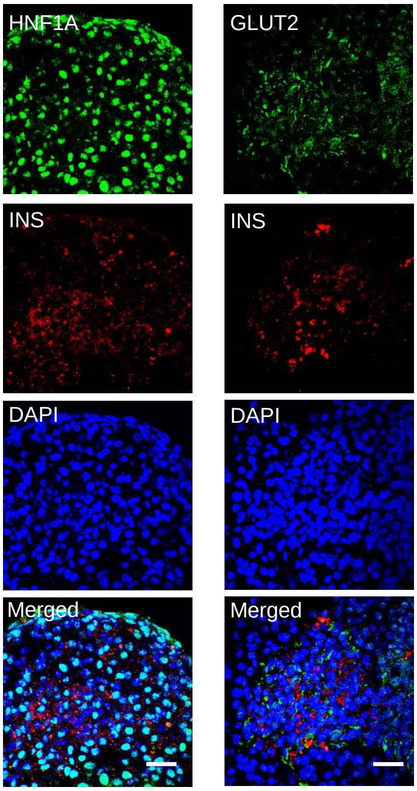

Supplementary Figure 9. Evaluation of HNF1A and GLUT2 co-expression with INS in β-like cells. Immunohistochemistry stain for HNF1A (green), GLUT2 (green), INS (red) proteins and nuclear stain using DAPI (blue) in cell clusters at day D35 of pancreatic differentiation. n = 3 independent experiments. (scale bar: 100 µm). D: day of differentiation.

Figure S10: Low et al.,

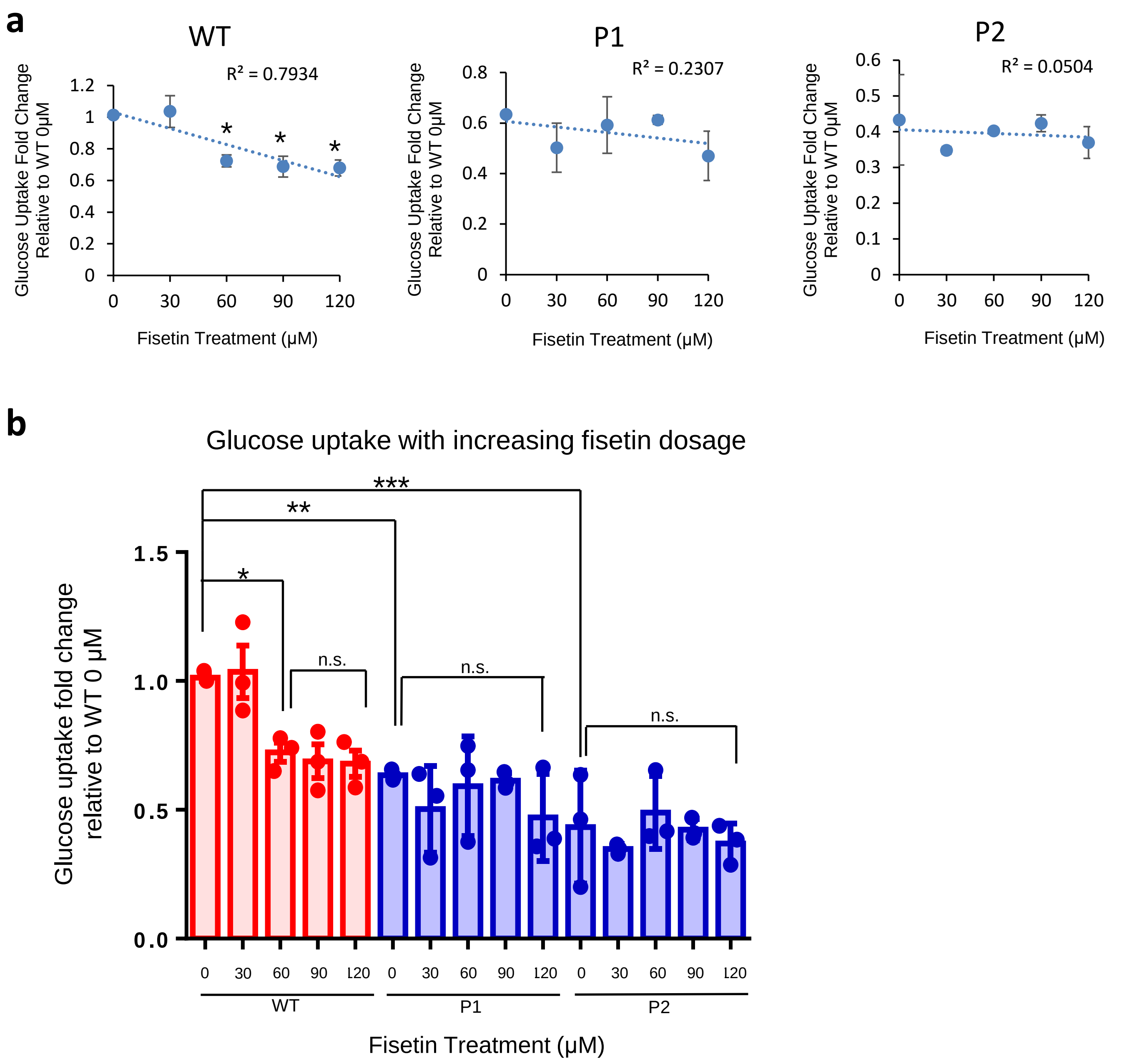

Supplementary Figure 10. Evaluation of increasing fisetin dosage on glucose uptake inhibition. (a) Glucose uptake fold changes with increasing concentrations (0 µm, 30 µm, 60 µm, 90 µm and 120 µm) of GLUT2 inhibitor, fisetin in WT (red), P1 and P2 (blue) hPSC-derived β-like cells. Effectiveness of fisetin in reducing glucose uptake measured by Pearson's R^2. Glucose uptake fold changes are normalized to glucose uptake amount in the presence of dimethyl sulfoxide (DMSO) (0 µm fisetin) in WT β-like cells. n = 3 independent experiments; *p* = 0.0098 (60 µm); 0.0341 (90 µm), 0.0172 (120 µm). * indicates P-value < 0.05 using pairwise t-test compared to 0 µm treatment. (b) Glucose uptake in WT, P1 and P2 hPSC-derived β-like cells in the presence of DMSO (0 µm fisetin) or increasing concentrations (30 µm, 60 µm, 90 µm and 120 µm) of GLUT2 inhibitor, fisetin. Glucose uptake fold changes are normalized to glucose uptake amount in the presence of DMSO (0 µm fisetin) in WT β-like cells. n = 3 independent experiments. One-way ANOVA was performed for group comparison ($p = 5.14 \times 10^{-7}$, F-critical = 8.52). Pairwise t-test was performed for pairwise comparison among independent groups; * *p* = 0.0098, ** *p* = 0.00003, *** *p* = 0.0429, n.s.: non-significant. WT: wild type, P1: patient 1, P2: patient 2. hPSCs: human pluripotent stem cells. For all statistical analysis: Error bars represent standard error of mean (SEM). Source data are provided as a Source data file.

Figure S11: Low et al.,

a

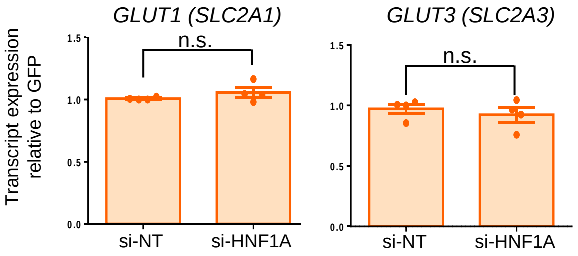

b

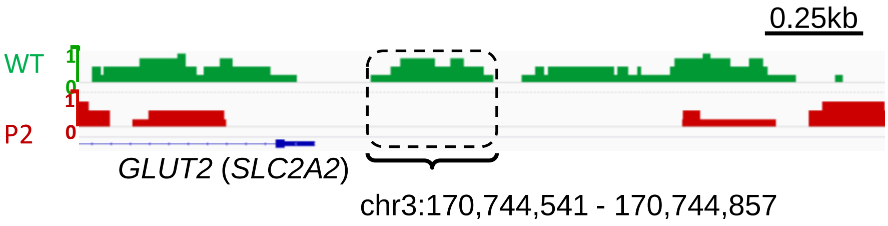

Supplementary Figure 11. Evaluation of GLUTs. (a) RT-qPCR analysis of *GLUT1* and *GLUT3* transcripts in EndoC-βH1 cells transfected with *HNF1A* siRNA (si-HNF1A) and non-targeting siRNA as negative control (si-NT) (n=3). For all statistical analysis: Error bars represent standard error of mean (SEM). Unpaired one-tailed Student's t-test was performed. n = 3 independent experiments. * indicates P-value < 0.05. (b) Histogram showing ChIP-Seq enrichment region in HNF1A-binding site on *GLUT2* (*SLC2A2*) promoter. Region where there is a difference in binding between WT (green) and P2 ($HNF1A^{+/H126D}$) (red) is highlighted in dotted-line box with the nearest coding gene shown. WT: wild type, P1: patient 1, P2: patient 2. RT-qPCR: quantitative reverse transcription polymerase chain reaction. ChIP-Seq: Chromatin immunoprecipitation sequencing. Source data are provided as a Source data file.
