# Supplementary material for: Decreased GLUT2 and glucose uptake contribute to insulin secretion defects in MODY3/HNF1A hiPSC-derived mutant β cells
Source: Nat Commun. 2021 May 25;12:3133. doi: 10.1038/s41467-021-22843-4 (PMC8149827; doi:10.1038/s41467-021-22843-4)
Supplement: Supplementary file 2 — Reporting Summary [file 41467_2021_22843_MOESM2_ESM.pdf]

nature research

Corresponding author(s): Adrian Kee Keong Teo

Last updated by author(s): Mar 23, 2021

Reporting Summary

Nature Research wishes to improve the reproducibility of the work that we publish. This form provides structure for consistency and transparency in reporting. For further information on Nature Research policies, see our Editorial Policies and the Editorial Policy Checklist.

Statistics

For all statistical analyses, confirm that the following items are present in the figure legend, table legend, main text, or Methods section.

n/a	Confirmed	
☐	☒	The exact sample size (*n*) for each experimental group/condition, given as a discrete number and unit of measurement
☐	☒	A statement on whether measurements were taken from distinct samples or whether the same sample was measured repeatedly
☐	☒	The statistical test(s) used AND whether they are one- or two-sided<br>*Only common tests should be described solely by name; describe more complex techniques in the Methods section.*
☒	☐	A description of all covariates tested
☒	☐	A description of any assumptions or corrections, such as tests of normality and adjustment for multiple comparisons
☐	☒	A full description of the statistical parameters including central tendency (e.g. means) or other basic estimates (e.g. regression coefficient) AND variation (e.g. standard deviation) or associated estimates of uncertainty (e.g. confidence intervals)
☐	☒	For null hypothesis testing, the test statistic (e.g. *F*, *t*, *r*) with confidence intervals, effect sizes, degrees of freedom and *P* value noted<br>*Give P values as exact values whenever suitable.*
☒	☐	For Bayesian analysis, information on the choice of priors and Markov chain Monte Carlo settings
☒	☐	For hierarchical and complex designs, identification of the appropriate level for tests and full reporting of outcomes
☐	☒	Estimates of effect sizes (e.g. Cohen's *d*, Pearson's *r*), indicating how they were calculated

Our web collection on statistics for biologists contains articles on many of the points above.

Software and code

Policy information about availability of computer code

Data collection	No software was used.
Data analysis	PRISM v8<br>FlowJo 7.0<br>Tophat v2.0.14<br>HTSeq v0.6.1p1<br>edgeR v3.1.3<br>Trimmomatic v0.36<br>bowtie2 v2.3.4.3<br>AMBER v 14<br>VMD v 1.9.1<br>PyMOL v 1.3<br>HOMER v 4.11<br>MUSCLE v 3.6<br>Axiovision LE v 4.9.1<br>Olympus Fluoview v 3.1<br>Microsoft Excel 2016

For manuscripts utilizing custom algorithms or software that are central to the research but not yet described in published literature, software must be made available to editors and reviewers. We strongly encourage code deposition in a community repository (e.g. GitHub). See the Nature Research guidelines for submitting code & software for further information.

Data

Policy information about availability of data

All manuscripts must include a data availability statement. This statement should provide the following information, where applicable:

- Accession codes, unique identifiers, or web links for publicly available datasets
- A list of figures that have associated raw data
- A description of any restrictions on data availability

Data reported in this paper were deposited into Gene Expression Omnibus (GEO) accessible via accession numbers GSE140208 (RNA-Seq) (https://www.ncbi.nlm.nih.gov/geo/query/acc.cgi?acc=GSE140208) and GSE139832 (ChIP-Seq) https://www.ncbi.nlm.nih.gov/geo/query/acc.cgi?acc=GSE139832). Crystal structure of WT HNF1A–DNA complex is available in Protein Data Bank (PDB 1IC8) (https://www.rcsb.org/structure/1ic8).

Field-specific reporting

Please select the one below that is the best fit for your research. If you are not sure, read the appropriate sections before making your selection.

☒ Life sciences ☐ Behavioural & social sciences ☐ Ecological, evolutionary & environmental sciences

For a reference copy of the document with all sections, see nature.com/documents/nr-reporting-summary-flat.pdf

Life sciences study design

All studies must disclose on these points even when the disclosure is negative.

Sample size	WT = sample size of 2 (H9, iAGb)<br>P1 = sample size of 3 (1a, 1b, 1c)<br>P2 = sample size of 3 (2a, 2b, 2c)<br>This is a biological study. We apply the rule of thumb of minimum n=3. As this is not a population study, size calculation was not performed.
Data exclusions	No data excluded.
Replication	All experiments were done at least 3 times and are reproducible.
Randomization	This is a prospective biological study. The study design does not require randomization.
Blinding	This is a prospective biological study. No blinding is applicable in this study.

Reporting for specific materials, systems and methods

We require information from authors about some types of materials, experimental systems and methods used in many studies. Here, indicate whether each material, system or method listed is relevant to your study. If you are not sure if a list item applies to your research, read the appropriate section before selecting a response.

Materials & experimental systems

n/a	Involved in the study	
☐	☒	Antibodies
☐	☒	Eukaryotic cell lines
☒	☐	Palaeontology and archaeology
☐	☒	Animals and other organisms
☐	☒	Human research participants
☒	☐	Clinical data
☒	☐	Dual use research of concern

Methods

n/a	Involved in the study	
☐	☒	ChIP-seq
☐	☒	Flow cytometry
☒	☐	MRI-based neuroimaging

Antibodies

Antibodies used	Goat anti-OCT4 Santa Cruz sc-8628 RRID:AB_653551<br>Rabbit anti-SOX2 Abcam ab97959 RRID:AB_2341193<br>Goat anti-NANOG R&D Systems AF1997 RRID:AB_355097<br>Mouse anti-SSEA4 STEMCELL Technologies 60062AD RRID:AB_2721031<br>Mouse anti-TRA-1-60 STEMCELL Technologies 60064AD RRID:AB_2686905<br>Mouse anti-ACTB (β-actin) Cell Signaling Technology 3700S RRID:AB_2242334<br>Rat anti-C-peptide Developmental Studies Hybridoma Bank GN-ID4 RRID:AB_2255626<br>Mouse anti-GATA4 Thermo Fisher Scientific MA5-15532 RRID:AB_10989032

Rabbit anti-GLUT2 Abcam ab95256 RRID:AB_10859605
Rabbit anti-GLUT2 Santa Cruz sc_9117 RRID:AB_641068
Rabbit anti-HNF1A Abcam ab96777 RRID:AB_10679303
Rabbit anti-HNF1A Abcam ab204306
Goat anti-HNF1A Santa Cruz sc-6547 RRID:AB_648295
Goat anti-HNF1A Santa Cruz sc-6548 RRID:AB_648293
Guinea pig anti-INS Abcam ab7842 RRID:AB_306130
Goat anti-NKX6.1 LifeSpan BioSciences LS-C124275 RRID:AB_10805839
Guinea pig anti-PDX1 Abcam ab47308 RRID:AB_777178
Goat anti-PDX1 R&D Systems AF2419 RRID:AB_355257
Alexa Fluor® 488 Invitrogen, A11055
Alexa Fluor ® 488 Invitrogen, 21202
Alexa Fluor® 488 Invitrogen, A21270
Alexa Fluor® 488 Invitrogen, A21206
Alexa Fluor® 594 Invitrogen, A11076
Alexa Fluor® 594 Invitrogen, A21203
Alexa Fluor® 594 Invitrogen, A11058
Alexa Fluor® 594 Invitrogen, A21207
Alexa Fluor® 488 Invitrogen, A21202
Alexa Fluor® 488 Invitrogen, A21206
Alexa Fluor® 647 Invitrogen, A21447
Alexa Fluor® 647 Jackson ImmunoResearch,706-605-148
anti-goat IgG-HRP Santa Cruz sc-2354
anti-mouse IgG-HRP Bethyl labs A90-516P

Validation

All antibodies are validated by the manufacturers.

Goat anti-OCT4 sc-8628): Epitope mapping near the N-terminus of Oct-3/4 of human origin.

Rabbit anti-SOX2 (ab97959)
Reacts with mouse, rat, human. Suitable for: Immunocytochemistry (ICC), Immunohistochemistry-paraffin (IHC-P), western blot (WB). ICC: NCCIT and NIH/3T3 cells. Dissociated induced pluripotent stem cells from mouse embryonic fibroblasts. Mouse embryonic stem cells. WB: NCCIT, IOUD2, HUES7, F9 and MCF7 whole cell lysate. IHC: Human brain glioma.

Goat anti-NANOG (AF1997)
Detects human Nanog in direct ELISAs and Western blots. In direct ELISAs, less than 1% cross-reactivity with recombinant mouse Nanog is observed. Application: western blot, chromatin immunoprecipitation, Immunocytochemistry.

Mouse anti-SSEA4 (60062AD)
Reactive Species: rhesus, cat, chicken, dog, human, mouse, rabbit, rat. Application: cell isolation, ELISA, flow cytometry, immunocytochemistry, immunofluorescence, immunohistochemistry.

Mouse anti-TRA-1-60 (60064AD)
Reactive Species: human, rhesus, rabbit. Application: cell isolation, flow cytometry, immunocytochemistry, immunofluorescence, immunoprecipitation, western blotting.

Mouse anti-ACTB (β-actin) (3700S)
Specificity / Sensitivity: detects endogenous levels of total β-actin protein. Due to the high sequence identity between the cytoplasmic actin isoforms, β-actin and cytoplasmic γ-actin, this antibody may cross-react with cytoplasmic γ-actin. It does not cross-react with α-skeletal, α-cardiac, α-vascular smooth, or γ-enteric smooth muscle isoforms. Species Reactivity: human, mouse, rat, hamster, monkey, dog. Application: western blotting, immunohistochemistry (paraffin), immunofluorescence (immunocytochemistry), flow cytometry.

Rat anti-C-peptide (GN-ID4)
Recognizes the C-peptide (aa 33-63 of proinsulin) which separates insulin B chain (aa 1-30) from insulin A chain (aa 66-86) in the proinsulin protein (minus signal peptide sequence). Stains C-peptide in mature granules and proinsulin in immature granules of islet beta-cells. The antibody does not cross-react with rodent C-peptide/proinsulin. Species Reactivity: human, monkey. Applications: FACS, immunofluorescence, immunohistochemistry, immunoprecipitation.

Mouse anti-GATA4 (MA5-15532)
Species Reactivity: human, mouse, rat. Applications: western blot, immunocytochemistry, ChIP assay.

Rabbit anti-GLUT2 (ab95256)
Specificity: recognizes the ~53-61 kDa GLUT2 protein. It does not recognize other GLUT isoforms. Tested applications: Immunocytochemistry-immunoflourescence. Species reactivity: Human.

Rabbit anti-GLUT2 (sc_9117)
Species reactivity: mouse, rat, human. Applications: western blotting, immunoprecipitation, immunofluorescence, immunohistochemistry, ELISA .

Rabbit anti-HNF1A (ab96777)
Reacts with: Human. Applications: western blotting, immunohistochemistry-paraffin, immunocytochemistry-immunoflourescence.

Rabbit anti-HNF1A (ab204306)
Species reactivity: Human. Tested applications: immunocytochemistry-immunoflourescence, immunohistochemistry-paraffin.

Goat anti-HNF1A (sc-6547)
Species reactivity: mouse, rat, human. Applications: western blotting, immunoprecipitation, immunofluorescence, ELISA.

Goat anti-HNF1A (sc-6548)
Raised against amino acids 80-284 of HNF-1α of human origin. Species reactivity: mouse, rat, human. Applications: western blotting, immunoprecipitation, immunoflourescence, ELISA.

Guinea pig anti-INS (ab7842)
Species reactivity: mouse, rat, human, hamster. Applications: flow cytometry/cell sorting, immunocytochemistry-immunoflourescence, immunohistochemistry, immunohistochemistry-paraffin, immunoprecipitation.

Goat anti-NKX6.1 (LS-C124275)
Species reactivity: human, mouse. Applications: immunohistochemistry, western blotting, ELISA.

Guinea pig anti-PDX1 (ab47308)
Species reactivity: Mouse. Applications: immunocytochemistry-immunoflourescence.

Goat anti-PDX1 (AF2419)
Species Reactivity: human. Applications: western blot, immunocytochemistry, immunohistochemistry

Eukaryotic cell lines

Policy information about cell lines

Cell line source(s)	H9 hESC line WiCell Research Institute NIHhESC-10-0062 RRID:CVCL_9773<br>AD-293 line Agilent 240085 RRID:CVCL_KA63<br>EndoC-βH1 line Univercell Biosolutions EndoC-βH1 RRID:CVCL_L909<br>Fibroblast (to generate iAGb) Coriell Institute AG16102 RRID:CVCL_2G48<br>CF-1 MEF Gibco A34181 RRID:CVCL_5251
Authentication	Authentication of hiPSCs were performed using karyotyping.
Mycoplasma contamination	All cell lines were tested negative for mycoplasma contamination.
Commonly misidentified lines (See ICLAC register)	No commonly misidentified cell lines were used in the study.

Animals and other organisms

Policy information about studies involving animals; ARRIVE guidelines recommended for reporting animal research

Laboratory animals	NOD-SCID mice Taconic Biosciences NOD/MrkBomTac-Prkdcscid RRID:IMSR_TAC:nodsc<br>Males, 4-8 weeks old
Wild animals	Study did not involve wild animals.
Field-collected samples	Study did not involve samples collected from the field.
Ethics oversight	A*STAR 2020-096; NHG DSRB 2013/01068.

Note that full information on the approval of the study protocol must also be provided in the manuscript.

Human research participants

Policy information about studies involving human research participants

Population characteristics	Human research participants are female, siblings, harbor the same heterozygous H126D HNF1A mutation, and diagnosed with diabetes at 12 years old. At the point of P1 and P2 patient recruitment, they were above 21 years old (adults) but the specific age at recruitment is not available.
Recruitment	Participants were recruited by A/P Lim Su Chi. A/P Lim is their attending physician for their monogenic diabetes condition. Their gDNA has been screened for monogenic diabetes and HNF1A mutation was identified.
Ethics oversight	NHG DSRB; A*STAR.

Note that full information on the approval of the study protocol must also be provided in the manuscript.

ChIP-seq

Data deposition

- [x] Confirm that both raw and final processed data have been deposited in a public database such as GEO.
- [ ] Confirm that you have deposited or provided access to graph files (e.g. BED files) for the called peaks.

Data access links<br>*May remain private before publication.*	We have deposited the BED files for the called peaks.<br>Data reported in this paper were deposited into Gene Expression Omnibus (GEO) accessible via accession numbers GSE140208 (RNA-Seq) (https://www.ncbi.nlm.nih.gov/geo/query/acc.cgi?acc=GSE140208) and GSE139832 (ChIP-Seq) https://www.ncbi.nlm.nih.gov/geo/query/acc.cgi?acc=GSE139832). Crystal structure of WT HNF1A–DNA complex is available in Protein Data Bank (PDB 1IC8) (https://www.rcsb.org/structure/1ic8).
Files in database submission	All files available are listed: https://www.ncbi.nlm.nih.gov/geo/query/acc.cgi?acc=GSE140208; https://www.ncbi.nlm.nih.gov/geo/query/acc.cgi?acc=GSE139832.
Genome browser session<br>(e.g. UCSC)	Not applicable.

Methodology

Replicates	Datasets in ChIP-Seq analysis use DNA samples from three replicates of HNF1A H126D iPSC-derived endocrine progenitors and two replicates of WT hPSC-derived endorcine progenitors.
Sequencing depth	The NEXTSEQ High Output was performed using the Illumina NEXTSEQ 500 Sequencers with the Illumina® Reagent v2 (75 cycle kit) Kit. The DNA was attached to the flowcell surfaces and amplified to clusters, followed by attachment with the Sequencing primers and run at 1x76cycles, generating Single-Read 75 base-pair reads.
Antibodies	Rabbit anti-HNF1A Abcam ab96777 RRID:AB_10679303
Peak calling parameters	Peaks were identified using the findPeaks program with default settings (-style factor).
Data quality	The quality of the reads is determined by Q-score calculation of the bcl2fastq software and attached PDF have the details. The data is only passed if the Q30 is above 75%, in this case all 3 libraries have passed.
Software	Trimmomatic v0.36<br>bowtie2 v2.3.4.3<br>Homer suite (UCSD)

Flow Cytometry

Plots

Confirm that:

- [x] The axis labels state the marker and fluorochrome used (e.g. CD4-FITC).
- [x] The axis scales are clearly visible. Include numbers along axes only for bottom left plot of group (a 'group' is an analysis of identical markers).
- [x] All plots are contour plots with outliers or pseudocolor plots.
- [x] A numerical value for number of cells or percentage (with statistics) is provided.

Methodology

Sample preparation	D20 endocrine progenitors and D34 beta-like cells were differentiated from hPSCs using pancreatic differentiation protocol
Instrument	BD FACSymphony™ Flow Cytometer A5
Software	FlowJo 7.0 software
Cell population abundance	No cell sorting was performed in this manuscript
Gating strategy	First differentiated pancreatic lineage cells were gated on FSC /SSC, followed by gating for single cells on FSH/FSC. Cells were stained for markers of interest using antibodies. Percentage of positive cells were gated against blank and negative (secondary only) control staining.

- [x] Tick this box to confirm that a figure exemplifying the gating strategy is provided in the Supplementary Information.
