# Supplementary material for: Decreased GLUT2 and glucose uptake contribute to insulin secretion defects in MODY3/HNF1A hiPSC-derived mutant β cells
Source: Nat Commun. 2021 May 25;12:3133. doi: 10.1038/s41467-021-22843-4 (PMC8149827; doi:10.1038/s41467-021-22843-4)
Supplement: Supplementary file 3 — Description of Additional Supplementary Files [file 41467_2021_22843_MOESM3_ESM.pdf]

Description of Additional Supplementary Files

File Name:Supplementary Movie 1
Description: Molecular dynamics simulation of unbound WT HNF1A.

File Name:Supplementary Movie 2
Description: Molecular dynamics simulation of unbound HNF1A H126D mutant.

File Name:Supplementary Data 1
Description: List of 1682 genes that were downregulated in mutant *HNF1A*$^{+/H126D}$ endocrine progenitors compared to WT cells. FC> 1.5, P-value ≤ 0.05. Adjusted P-values were determined using Fisher's exact test according to the edgeR protocol (Robinson et al., 2010).

File Name:Supplementary Data 2
Description: List of 512 genes that were upregulated in mutant *HNF1A*$^{+/H126D}$ endocrine progenitors compared to WT cells. FC> 1.5, P-value ≤ 0.05. Adjusted P-values were determined using Fisher's exact test according to the edgeR protocol (Robinson et al., 2010).

File Name:Supplementary Data 3
Description: List of 347 common genes that were downregulated in *HNF1A*$^{+/H126D}$ in endocrine progenitors and in *HNF1A*$^{+/T260M}$ MODY3 islets. FC> 1.5, P-value ≤ 0.05. Adjusted P-values were determined using Fisher's exact test according to the edgeR protocol (Robinson et al., 2010).

File Name:Supplementary Data 4
Description: List of 22 common genes that were downregulated in *HNF1A*$^{+/H126D}$ endocrine progenitors and in *HNF1A*$^{+/-}$ hESC-derived β cells. FC> 1.5, P-value ≤ 0.05. Adjusted P-values were determined using Fisher's exact test according to the edgeR protocol (Robinson et al., 2010).

File Name:Supplementary Data 5
Description: List of 394 binding regions from 374 gene targets bound by WT HNF1A but not by H126D mutant in endocrine progenitors.
